# Basidiomycetes Are Particularly Sensitive to Bacterial Volatile Compounds: Mechanistic Insight Into the Case Study of *Pseudomonas protegens* Volatilome Against *Heterobasidion abietinum*

**DOI:** 10.3389/fmicb.2021.684664

**Published:** 2021-05-31

**Authors:** Maria Isabella Prigigallo, Angelo De Stradis, Abhishek Anand, Francesco Mannerucci, Floriane L’Haridon, Laure Weisskopf, Giovanni Bubici

**Affiliations:** ^1^Istituto per la Protezione Sostenibile delle Piante, Consiglio Nazionale delle Ricerche, Bari, Italy; ^2^Department of Biology, University of Fribourg, Fribourg, Switzerland; ^3^Dipartimento di Scienze del Suolo, della Pianta e degli Alimenti, Università degli Studi di Bari Aldo Moro, Bari, Italy

**Keywords:** *Heterobasidion*, *Pseudomonas*, volatile organic compounds (VOCs), biological control agents, biocontrol, microbe–microbe interaction, antagonism

## Abstract

Volatile organic compounds (VOCs) play an important role in the communication among organisms, including plants, beneficial or pathogenic microbes, and pests. *In vitro*, we observed that the growth of seven out of eight Basidiomycete species tested was inhibited by the VOCs of the biocontrol agent *Pseudomonas protegens* strain CHA0. In the Ascomycota phylum, only some species were sensitive (e.g., *Sclerotinia sclerotiorum*, *Botrytis cinerea*, etc.) but others were resistant (e.g., *Fusarium oxysporum* f. sp. *cubense*, *Verticillium dahliae*, etc.). We further discovered that CHA0 as well as other ten beneficial or phytopathogenic bacterial strains were all able to inhibit *Heterobasidion abietinum*, which was used in this research as a model species. Moreover, such an inhibition occurred only when bacteria grew on media containing digested proteins like peptone or tryptone (e.g., Luria-Bertani agar or LBA). Also, the inhibition co-occurred with a pH increase of the agar medium where the fungus grew. Therefore, biogenic ammonia originating from protein degradation by bacteria was hypothesized to play a major role in fungus inhibition. Indeed, when tested as a synthetic compound, it was highly toxic to *H. abietinum* (effective concentration 50% or EC_50_ = 1.18 M; minimum inhibitory concentration or MIC = 2.14 M). Using gas chromatography coupled to mass spectrometry (GC/MS), eight VOCs were found specifically emitted by CHA0 grown on LBA compared to the bacterium grown on potato dextrose agar (PDA). Among them, two compounds were even more toxic than ammonia against *H. abietinum*: dimethyl trisulfide had EC_50_ = 0.02 M and MIC = 0.2 M, and 2-ethylhexanol had EC_50_ = 0.33 M and MIC = 0.77 M. The fungus growth inhibition was the result of severe cellular and sub-cellular alterations of hyphae occurring as early as 15 min of exposure to VOCs, as evidenced by transmission and scanning electron microscopy observations. Transcriptome reprogramming of *H. abietinum* induced by CHA0’s VOCs pointed out that detrimental effects occurred on ribosomes and protein synthesis while the cells tried to react by activating defense mechanisms, which required a lot of energy diverted from the growth and development (fitness cost).

## Introduction

The Basidiomycota phylum comprises about 30,000 species of mushrooms, yeasts, rust, and smut fungi. They are ubiquitous in terrestrial ecosystems where they are the major wood-degrading fungi (Kirk et al., 2001; Mitchell, 2005; Hibbett, 2006). As mycorrhizal symbionts, Basidiomycetes are important for forest health and, in the evolutionary history, they may have been vital to land colonization by plants (Pirozynski and Malloch, 1975; Smith and Read, 2008; Feijen et al., 2018). Around 8,000 rust and smut species are plant pathogens, and at least 40 Basidiomycetes cause disease in mammals (e.g., *Cryptococcus*) (Cheng et al., 2001; Kirk et al., 2001; Mitchell, 2005; Heitman, 2011; Bakkeren et al., 2012). The Basidiomycetes *Puccinia* spp., *Ustilago maydis*, and *Melampsora lini* have been considered among the top ten fungal plant pathogens based on their perceived importance, either scientific or economical (Dean et al., 2012). They are responsible for epidemics on widely grown crops such as cereals, hence their control requires large amounts of fungicides (Bakkeren et al., 2012). *Rhizoctonia solani* is another Basidiomycete with a worldwide relevance as a plant pathogen (Anderson, 1982; Garcia et al., 2006). Disease-suppressive soils have been known and largely studied for Rhizoctonia root rot (Jambhulkar et al., 2015; Schlatter et al., 2017). The microbiota has been correlated with such a suppressiveness of the soil, suggesting that beneficial microbes may play an important role in the management of Rhizoctonia diseases (Cordovez et al., 2015; Jambhulkar et al., 2015; Schlatter et al., 2017). Nevertheless, despite several experimental proofs (D’Aes et al., 2011; Huang et al., 2012; de Tenório et al., 2019; Wu et al., 2019), there are no examples of large-scale biological control of this pathogen. On the other hand, a spectacular example of biological control is the use of *Phlebiopsis gigantea* against *Heterobasidion* spp., which are both Basidiomycetes. *Heterobasidion annosum sensu lato* (s.l.) is a cosmopolitan pathogen causing tree mortality and wood decay of economic importance in coniferous forests (Woodward et al., 1998). Every year, around 35,000 ha of forest in Sweden are treated with *P. gigantea* (Rotstop^®^, Verdera, Finland) (Thor, 2003), indicating that it is a concrete control means amongst those available against *Heterobasidion* spp. (Nicolotti and Gonthier, 2005; Garbelotto and Gonthier, 2013). Hence, beneficial microbes are valuable control means against Basidiomycetes. Several other products have been successfully used to treat pine or spruce stumps, including sodium tetraborate decahydrate (borax) and urea (Pratt et al., 1998; Gonthier and Thor, 2013; Gonthier, 2019).

During routine experiments in our laboratory, we observed that *Pseudomonas protegens* strain CHA0 inhibited *H. abietinum* growth through volatile compounds. Biological control agents act by multiple mechanisms, and volatile organic compounds (VOCs) have gained increasing interest in the last decades because, besides their role in biocontrol, they also participate in the cross-talk between microbes and other organisms in the environment (Weisskopf et al., 2016). Several compounds such as hydrogen cyanide (HCN), ammonia (NH3), 1-undecene, dimethyl disulfide, dimethyl trisulfide, and other volatiles have been identified in bacteria over years and often associated with the biocontrol of diverse plant pathogens (De Vrieze et al., 2015 and references therein; Giorgio et al., 2015; Hunziker et al., 2015; Ossowicki et al., 2017; Guevara-Avenda[~no et al., 2019; Báez-Vallejo et al., 2020). Remarkably, dimethyl disulfide has found its application in agriculture as a novel soil fumigant (Wang et al., 2009; Leocata et al., 2014; Mao et al., 2019; Yan et al., 2019).

This research was aimed at investigating the effects of bacterial VOCs against Basidiomycetes, using *P. protegens* CHA0 and *H. abietinum* as model species. *Pseudomonas protegens* CHA0 possesses several biocontrol traits (HCN, 2,4-diacetylphloroglucinol, pyoluteorin, and pyrrolnitrin) as well as a gene cluster (*fit*) with insecticidal activity (Haas and Défago, 2005; Péchy-Tarr et al., 2008). It is the type strain of the species (Ramette et al., 2011) and its genome was completely sequenced in 2014 (Jousset et al., 2014). The objectives of this study were: (a) evaluation of the effectiveness of bacterial VOCs against diverse fungi and oomycetes, especially *H. abietinum*; (b) identification of the effective CHA0’s volatiles; and (c) study of the mode of action of the CHA0’s volatilome against *H. abietinum*.

Therefore, through several *in vitro* experiments, we described the effects of CHA0’s VOCs against diverse fungi and oomycetes, the effects of VOCs emitted by phylogenetically distant bacteria against *H. abietinum*, and the importance of the substrate composition in eliciting the emission of effective volatiles. Then, we assessed the involvement of hydrogen cyanide and ammonia as volatiles toxic to *H. abietinum*. Also, using gas chromatography coupled to mass spectrometry (GC/MS), we identified the CHA0’s VOCs differentially produced on Luria-Bertani agar (LBA) and potato dextrose agar (PDA), i.e., two media selected as contrasting conditions supporting or not, respectively, the production of bacterial volatiles effective against *H. abietinum.* Finally, these two contrasting conditions were used to observe the cellular and sub-cellular injuries induced by CHA0’s volatiles in *H. abietinum* using transmission and scanning electron microscopy, and the corresponding transcriptional re-programming through transcriptomics (RNA-Seq).

## Materials and Methods

### Overlapping Plate Assay

The overlapping plate (OP) assay was used for all the experiments aimed at evaluating the effectiveness of bacterial VOCs in inhibiting fungal growth (Giorgio et al., 2015). Bacteria tested for the production of VOCs were grown for 2 days in a Petri plate containing an agar medium (e.g., LBA, PDA, peptone agar or PA, King’s B agar or KBA, etc.). Inoculation of plates was made by plating with a glass rod 100 μL suspension of 10^7^ cells mL^–1^ obtained from a 24 h-culture in LB broth (250 rpm, 27°C). Fungi were inoculated in another Petri plate containing PDA using a 6 mm-agar plug taken from the edge of an actively growing colony (5-day-old PDA culture). The plate with the fungus was put upside down on top of the plate with the bacterium, both without the lid. The plates were sealed with Parafilm^®^ M to avoid the escape of VOCs from the headspace of the bacteria and fungi. Plates were grown in triplicate and incubated in the dark at 27°C before and after the sealing. At the end of the experiment, i.e., 7 or 10 days, the fungal colony diameter was measured to calculate the growth inhibition compared to control.

For scanning electron microscopy observations and transcriptomics analysis, the OP assay was established with one modification: the fungus was not grown directly on PDA, but on an autoclaved cellophane disk (100 mm) placed onto PDA. This allowed to collect easily the mycelium from the cellophane for RNA extraction and to transfer intact mycelium for microscopic observations by cutting squares of cellophane supporting the fungal growth.

### Effects of CHA0’s VOCs Against Several Fungal Species

*Experiment A*. Using the OP assay, the effects of VOCs produced by *P. protegens* strain CHA0 grown on LBA were tested against several fungal species belonging to Basidiomycota (*H. abietinum*, *H. parviporum*, *Fomes fomentarius*, *Ganoderma lucidum*, *Phellinus pini*, *P. tuberculosus*, *Daedalea quercina*, and *Rhizoctonia solani*), Ascomycota (*Aspergillus* sp., *Penicillium* sp., *Fusarium oxysporum* f. sp. *cubense*, *Verticillium dahliae*, *Pyrenochaeta lycopersici, Alternaria tomatophila*, *Botrytis cinerea*, and *Sclerotinia sclerotiorum*), Oomycota (*Pythium aphanidermatum*), and Zygomycota (*Rhizopus* sp.). Plates with LBA without CHA0 were used as a negative control. The experiment duration was 7 days.

### Effects of VOCs Produced by Several Bacteria Against *H. abietinum*

*H. abietinum* strain 10 was used as a model Basidiomycete fungus in all the successive experiments. It was isolated in 2007 from a diseased silver fir (*Abies alba* Mill.) at Marsico Nuovo (Potenza province, in the Basilicata region, Italy), and identified based on microscopic characteristics and multilocus sequence analysis (Dalman et al., 2010) (Supplementary Table 1 and Supplementary Figure 1), using sequence data generated with the RNA-Seq experiment. This strain was stored in the collection of the ‘Dipartimento di Scienze del Suolo, della Pianta e degli Alimenti’ at the University of Bari Aldo Moro.

*Experiment B.* To evaluate whether the *H. abietinum* growth inhibition was a trait specific to *P. protegens* CHA0, several bacteria were grown on LBA or PDA and tested as described above (OP assay): three *Pseudomonas* spp. isolates, three *Bacillus* spp. isolates (isolated from the soil), the laboratory strain *Escherichia coli* TG1, and three plant pathogens such as *Pectobacterium carotovorum* subsp. *carotovorum* NCPPB 312 (type strain), *Pseudomonas syringae* pv. *tomato*, and *Agrobacterium tumefaciens* biovar 1 isolate 396.

### Effects of VOCs on Differently Aged Mycelium

*Experiment C.* With the hypothesis that young hyphae could be more sensitive than the old ones to bacterial VOCs, colonies of *H. abietinum* strain 10 of different ages were exposed to CHA0’s VOCs in an OP assay as reported above. CHA0 was grown on LBA for 2 days, while *H. abietinum* strain 10 was grown on PDA for 3, 5, 7 days before joining and sealing the plates, or inoculated at the moment (0) of plate joining.

Another experiment, Supplementary Experiment 1, was established to assess the magnitude of CHA0’s effect against *H. abietinum* growth.

### Role of Hydrogen Cyanide (HCN) and Effects of Different Agar Media on the Production of VOCs

*Experiment D.* To assess the involvement of hydrogen cyanide (HCN) in the *H. abietinum* growth inhibition, the strain CHA77, which is the HCN-knockdown mutant of CHA0, was tested. The production of HCN by CHA0 and CHA77 was preliminary ascertained according to two methods (Supplementary Experiment 2), one based on copper(II) ethylacetoacetate and 4,4′-methylenebis-(*N*,*N*-dimethylaniline) (Castric and Castric, 1983; Voisard et al., 1989), and another based on picric acid and sodium carbonate (Bakker and Schippers, 1987).

Since NaCl and glycine have been known to stimulate HCN production by bacteria (Castric, 1977; Blumer and Haas, 2000), the amendment of agar media with 10 g L^–1^ NaCl and 4.4 g L^–1^ glycine was also tested. Also, to evaluate whether the agar medium affected the production of HCN and other VOCs, *P. protegens* strains CHA0 and CHA77 were grown on LBA, PDA, peptone-agar (PA), or King’s B agar (KBA), with or without NaCl and glycine, and tested against *H. abietinum* strain 10 grown on PDA as described above (OP assay). Therefore, a total of nine media (including an LBA without NaCl as well) were used.

Luria-Bertani agar was prepared as follows: 10 g L^–1^ tryptone (Oxoid, LP0042), 5 g L^–1^ yeast extract (Oxoid, LP0021), 5 g L^–1^ NaCl, 15 g L^–1^ agar (Oxoid, LP0011, agar bacteriological No. 1). PDA was prepared as follows: 39 g L^–1^ potato dextrose agar (Conda, cat. no. 1022). PA was prepared as follows: 10 g L^–1^ Bacto^TM^ proteose peptone (Becton Dickinson, ref. 211684), 20 g L^–1^ agar (Oxoid, LP0011, agar bacteriological No. 1). KBA was prepared as follows: 20 g L^–1^ Bacto^TM^ proteose peptone (Becton Dickinson, ref. 211684), 1.5 g L^–1^ K_2_HPO_4_, 1.5 g L^–1^ MgSO_4_⋅7 H_2_O, 20 g L^–1^ agar (Oxoid, LP0011, agar bacteriological No. 1), final pH 7.2 at 25°C.

The experiment duration was 10 days.

### Effect of Ammonia and pH on *H. abietinum*

The production of ammonia by CHA0, CHA77, and other bacteria was preliminarily tested in several growth media using Nessler’s reagent as described in Supplementary Experiment 3.

*Experiment E.* To assess the participation of ammonia in the fungal growth inhibition, CHA0 and *H. abietinum* strain 10 were co-cultured with phosphoric acid (H_3_PO_4_). Phosphoric acid reacts with ammonia to form ammonium phosphate salts (Ryden and McNeill, 1984). The experiment was performed in 150 mm Petri plates where three 55 mm Petri plates (*a*, *b* and *c*) were placed. Plate *a* contained PDA and was inoculated with 6 mm agar plug of *H. abietinum* strain 10; the plate *b* contained LBA and was inoculated with five CHA0 colonies, while a non-inoculated plate served as control; the plate *c* contained 1 M phosphoric acid, while sterile distilled water was used for the control. Therefore, all combinations of the 55 mm plates were arranged in a total of four 150 mm plates, which were sealed with Parafilm^®^ M to avoid the VOCs escape. This experimental setup was conducted in triplicate and incubated at 27°C for 7 days. At the end of the experiment, a piece of litmus paper was placed on the agar surface to check its pH.

*Experiment F.* To test whether bacteria can alkalize an agar medium at distance by their emission of ammonia and whether *H. abietinum* strain was affected by pH an OP assay was carried out with CHA0 and CHA77 grown on LBA or PDA, and *H. abietinum* strain 10 grown on PDA, unbuffered or buffered at pH 6.4 or 8.0. Buffered PDA was prepared using 0.1 M potassium phosphate buffers: pH 6.4 was obtained with 4.84 g L^–1^ K_2_HPO_4_ + 9.83 g L^–1^ KH_2_PO_4_, and pH 8 with 16.37 g L^–1^ K_2_HPO_4_ + 0.82 g L^–1^ KH_2_PO_4_ (Sambrook and Russel, 2001). At the end of the experiment, a piece of litmus paper was placed on the agar surface to check its pH.

### Identification of VOCs by Gas Chromatography Coupled to Mass Spectrometry (GC/MS)

*Experiment G.* CHA0 and CHA77 bacterial colonies were grown overnight in LB broth at 28°C with shaking at 180 rpm. Overnight cultures were then adjusted to OD_595_ = 1.0 and 100 μL suspension was spread onto 5 cm-glass plates with LBA or PDA. The plates were incubated at room temperature for 16 h before collecting volatiles for 48 h using the closed-loop stripping method with a charcoal filter. The charcoal filter was then washed with dichloromethane (VWR) to collect the trapped volatiles (Hunziker et al., 2015). Samples were then injected into a gas chromatograph coupled with a mass spectrometer (GC/MS; HP6890). An HP-5ms fused silica capillary column (30 m, 0.25 mm inner diameter, 0.25 μm film; Agilent Technologies) was used for this analysis. Conditions used to separate compounds were: 67 kPa inlet pressure, 15 mL min^–1^ He, 2 μL injection volume, 300°C transfer line, 250°C injector, 70 eV electron energy. The acquired raw MS data were converted into netCDF format using OpenChrom^®^ software (Wenig and Odermatt, 2010) and pre-processed using MZmine-2.20 (Pluskal et al., 2010) to create m/z and peak intensity table. This table was used as an input file for the statistical analysis performed using Metaboanalyst 4.0 (Xia et al., 2015). The data were filtered using the interquartile range (IQR) and normalized with log-transformation and automatic scaling. Data were subjected to the one-way analysis of variance (ANOVA), *post hoc* test (*P* < 0.05), hierarchical clustering (Euclidean distance and Ward’s linkage clustering algorithm), and principal component analysis (PCA). Significant mass features were identified (*post hoc* test; *P* < 0.05) and used to generate a list of compounds differentially emitted by CHA0 on LBA and PDA. The identification of compounds was done using the OpenChrom^®^ software coupled with the AMDIS and NIST 17 database.

### Testing Synthetic VOCs Against *H. abietinum*

*Experiment H.* Ammonia and the VOCs identified in Experiment G (Table 1) were tested *in vitro* against *H. abietinum* strain 10. Ammonia was tested as well, using ammonium hydroxide (NH_4_OH; CAS no. 1336-21-6; Carlo Erba Reagents). PDA Petri plates were inoculated with a 6 mm-agar plug of the fungal mycelium and positioned upside down. The volatile substances were poured in a watch glass placed on the plate lid before sealing the plates with Parafilm^®^ M. All the liquid substances were tested at 0.001, 0.01, 0.1, 0.2, 1, 2, 10, 20, 50, and 100 μL (per plate), and 3-phenylpropiophenone, which was the only compound in powder form, was tested at 0.001, 0.01, 0.1, 0.2, 1, 2, 10, 20, 50, and 100 mg (per plate). Amounts smaller than 10 μL or 10 mg were diluted in dimethyl sulfoxide (DMSO; CAS no. 67-68-5), or water for 3-phenylpropiophenone because insoluble in organic solvents, to improve handling and reduce technical errors; then, 100 μL DMSO- or water-solutions were poured in the watch glass. All the substances were tested in triplicate, and plates containing watch glasses with water, DMSO, or empty served as controls. Plates were incubated for 7 days at 27°C and, at the end of the experiment, a piece of litmus paper was placed on the agar surface to check its pH.

**TABLE 1:** *Experiment G.* Volatile compounds differentially produced by *Pseudomonas protegens* CHA0 grown on Luria-Bertani agar (LBA) compared to the same bacterium grown on potato dextrose agar (PDA) and to the non-inoculated media.

Compound name [IUPAC name]*^*a*^*	CAS number	Retention time (min)	Mass/charge ratio (m/z)	Probability (%)
1,3-Diphenylpropane [3-phenylpropylbenzene]	1081-75-0	28.35	92.05 91.05 196.05 105.05 43.95.	94.59
Dimethyl trisulfide [(methyltrisulfanyl)methane]	3658-80-8	9.08	125.95 44.05 44.95 78.95 58.05.	90.96
3-Phenylpropiophenone [1,3-diphenylpropan-1-one]	1083-30-3	32.63	105.05 210.05 77.05 91.05 51.05.	82.31
Acetonanil [2,2,4-trimethyl-1H-quinoline]	147-47-7	23.45	158.05 43.95 159.05 157.05 115.05.	68.92
2-Ethylhexanol [2-ethylhexan-1-ol]	104-76-7	11.33	57.05 41.05 43.05 43.95 55.05.	66.64
Acetophenone [1-phenylethanone]	98-86-2	12.50	105.05 77.05 120.05 51.05 50.05.	66.33
Phenol	108-95-2	9.66	94.05 66.05 65.05 39.05 40.05.	62.56
Butyldiglycol [2-(2-butoxyethoxy)ethanol]	112-34-5	16.48	57.05 45.05 41.05 59.05 43.05.	62.28
Unknown	–	15.23	78.05 106.05 135.95 51.05 77.05.	41.09
Unknown	–	44.68	207.05 91.05 129.05 206.05 105.05.	33.24
Unknown	–	27.18	71.05 43.05 57.05 43.95 85.05.	5.84

*^a^ Compounds were identified based on a comparison of the mass spectrum to a database spectrum (NIST 17 library).*

Data were used to determine the minimum inhibitory concentration (MIC) that inhibits fungal growth by 100%, and calculate by interpolation the effective concentration 50% (EC_50_), i.e., the compound quantity that reduces the colony diameter by 50% compared to control. MIC and EC_50_ were expressed both as microliters of VOC per Petri plate and molarity of the compound relative to the air space above the agar surface in the Petri plate, which was estimated to be ca. 40 mL.

Using this procedure, another experiment (Experiment I) was carried out to test the most effective volatiles, i.e., ammonia and dimethyl trisulfide, against five additional fungi such as *R. solani* (a Basidiomycete which, in Experiment A, was sensitive to CHA0’s VOCs like *H. abietinum*), *S. sclerotiorum*, *B. cinerea* (two sensitive Ascomycetes), *V. dahliae*, and *F. oxysporum* f. sp. *cubense* (two resistant Ascomycetes).

### Scanning Electron Microscopy (SEM)

*Experiment J.* The OP assay with cellophane agar was used for SEM observations. The mycelium of *H. abietinum* strain 10 exposed to the VOCs of *P. protegens* strain CHA0 grown on LBA was compared to the mycelium of the same fungal strain exposed to the VOCs of CHA0 grown on PDA (i.e., LBA vs. PDA). CHA0 was grown on LBA or PDA for 2 days, and *H. abietinum* strain 10 was grown on PDA for 5 days, both at 27°C in the dark. Then, the bacterium and fungal plates were joined without the lid and sealed, so the exposure to VOCs started. SEM inspections were made at 15 and 30 min, 1, 3, 6, 12, 24, 48, and 168 h of exposure to VOCs. Three replicates (plates) per treatment and time point were used, and three cellophane squares per plate were sampled at each time point. Cellophane squares were taken from the fungal colony edge for observing the actively growing hyphae. Cellophane squares were placed firmly onto aluminum stubs for SEM by double-sided carbon tape and directly subjected to gold/palladium sputter coating (Edwards Sputter Coater) under 10^–1^ mbar gas pressure and 20 mA current for 1 min. Coated samples were placed in the chamber of an SEM Hitachi TM 3000 Tabletop Microscope and observed in Standard Mode Observation with 15KV voltage supply and 5 × 10^–3^ mbar gas pressure.

### Transmission Electron Microscopy (TEM)

*Experiment K.* An experiment was established like for the SEM scrutiny (LBA vs. PDA), but the OP assay without cellophane was used. Observations were made at 15 min, 6 h, 12 h, and 24 h of exposure (*moe* or *hoe*, respectively) to CHA0’s VOCs. Three replicates (plates) per time point were used, and three PDA squares per plate were sampled at each time point. PDA squares were taken from the fungal colony edge for observing the actively growing hyphae and processed according to embedding standard procedures (Martelli and Russo, 1984). Briefly, mycelium was fixed in 4% glutaraldehyde in 0.05 M potassium phosphate buffer (pH 7.2) for 2 h and then it was post-fixed at 4°C in 1% osmium tetroxide in the same buffer for 2 h. Overnight bulk staining in 0.5% aqueous uranyl acetate, dehydration in graded ethanol dilutions, and embedding in TAAB Spurr resin followed. Longitudinal and cross ultra-thin sections (80 nm-thick), obtained by the ultra-microtome Reichert Supernova (Leica Reichert Division, Austria) on a Microstar diamond knife (Micro Engineering, Huntsville, AL, United States), were stained with lead citrate before observations with a Philips Morgagni 282D (FEI Company, Hillsboro, OR, United States) transmission electron microscope at 80 kV accelerating voltage. At least 20 sections per sample were inspected.

### Transcriptome Reprogramming of *H. abietinum* Investigated by RNA-Seq

*Experiment L.* Transcriptional changes of *H. abietinum* strain 10 were studied by RNA-Seq at 12 h of exposure to CHA0’s VOCs from LBA or PDA (LBA vs. PDA). An OP assay with cellophane agar was established with the experimental design used for the SEM observations. Three replicates (plates) were used. For RNA extraction, the entire mycelium of *H. abietinum* strain 10 colonies was scraped from the cellophane, transferred rapidly to a mortar containing liquid nitrogen, and ground with a pestle. RNA was extracted from 100 mg pulverized mycelium using the Quick-RNA Fungal/Bacterial Miniprep Kit (Zymo Research, CA, United States) and subjected to DNase digestion using TURBO DNA-free^TM^ Kit (Thermo Fisher Scientific, MA, United States). RNA was quantified with NanoDrop 1000 (Thermo Fisher Scientific) and the quality checked by gel electrophoresis before sending it to Macrogen Europe (The Netherlands) for library preparation and sequencing. Libraries were constructed using the TruSeq Stranded Total RNA with Ribo-Zero H/M/R_Gold kit (Illumina, San Diego, CA, United States) and 30 million reads of 101 bp per sample were sequenced with a paired-end mode in a NovaSeq system (Illumina, San Diego, CA, United States).

Data analysis was carried out using the Galaxy v. 19.01 platform (Afgan et al., 2018) locally installed on a computer equipped with 16 core-CPU and 64 GB RAM. Raw data were subjected to quality check and filtering using FASTQC v. 0.72+^1^ and Filter FASTQ tool v. 1.1.1 (Blankenberg et al., 2010), respectively: reads containing bases with a Phred quality score lower than 20 were discarded. The transcriptome of *H. abietinum* strain 10 was *de novo* assembled using Trinity (Galaxy version 2.8.4) (Grabherr et al., 2011) that yielded 12,459 transcripts with N50 of 1,204 bp for a total of 10,759,988 bp. Since this transcriptome was more fragmented than the transcriptome of *H. annosum* strain TC 32-1 v. 2.0 (Olson et al., 2012), which was already available at JGI Genome Portal (Project ID 16080^2^) (Nordberg et al., 2014), this latter was used as the reference genome for mapping the sequencing reads using TopHat2 (Galaxy version 2.1.1) (Kim et al., 2013). Quantification of transcript abundance was accomplished with featureCounts (Galaxy v. 1.6.4 + galaxy1) using only fragments with both reads aligned and with a paired-end distance between 50 and 600 bp; this was a stringent condition that yielded sound count data. Differentially expressed genes (DEGs) were identified with edgeR (Galaxy version 3.24.1 + galaxy1) with a statistical threshold of false discovery rate (FDR) < 0.05 (Robinson et al., 2009; Liu et al., 2015). No cut-off was applied to the gene expression fold change to select DEGs.

Gene ontology (GO) of proteins was retrieved from the JGI Genome Portal and additional annotations (e.g., GO and InterPro terms) were obtained with InterProScan 5 (Jones et al., 2014). Protein sequences and annotations were imported into Blast2GO v. 5.2.5 for the enrichment analysis by Fisher’s exact test (FDR < 0.05) and the construction of hierarchical graphs of the three GO categories such as biological process, molecular function, and cellular component (Conesa et al., 2005).

The enrichment of KEGG pathways and modules was also tested. For this purpose, mRNA sequences were converted into NCBI accessions by BLAST search and then converted to UniProt accession by DAVID 6.8 web service (Huang et al., 2009a, b). The mRNA sequence list converted to the UniProt accession list was subjected to the R/Bioconductor package clusterProfiler v. 3.12.0 for the KEGG pathway/module enrichment analysis using the *H. irregulare* database (hir) (Yu et al., 2012). The R/Bioconductor package pathview v. 1.24.0 was used to generate graphs showing genes and their expression levels placed onto KEGG pathways (Luo and Brouwer, 2013).

Finally, the expression level of the genes associated with the enriched GO and KEGG pathways/modules was plotted using the R package ggplot2 v. 3.3.2 (Wickham, 2016).

### Experimental Design and Statistical Analysis

All the experiments with Petri plates were conducted under a completely randomized design with three replicates (plates). Plates were incubated in the dark at 27°C. At the end of the experiments, i.e., 7 or 10 days, the fungal colony diameter was measured to calculate the growth inhibition compared to control. Data were subjected to the analysis of variance (ANOVA) and the means were compared using Fisher’s least significant difference (LSD) test. Before ANOVA, the normality of distribution and homoscedasticity were ascertained with Shapiro-Wilk’s test and Bartlett’s test, respectively. These statistical analyses were done with the software R ver. 4.0.2 (ISBN 3-900051-07-0^3^) within RStudio ver. 1.3.1056^4^. Plots were generated using the ggplot2 package ver. 3.3.2 (Wickham, 2016).

## Results

### Most Basidiomycetes Are Highly Sensitive to VOCs of *P. protegens* CHA0 While the Resistance Trait Is More Frequent in Ascomycota

**FIGURE 1:**
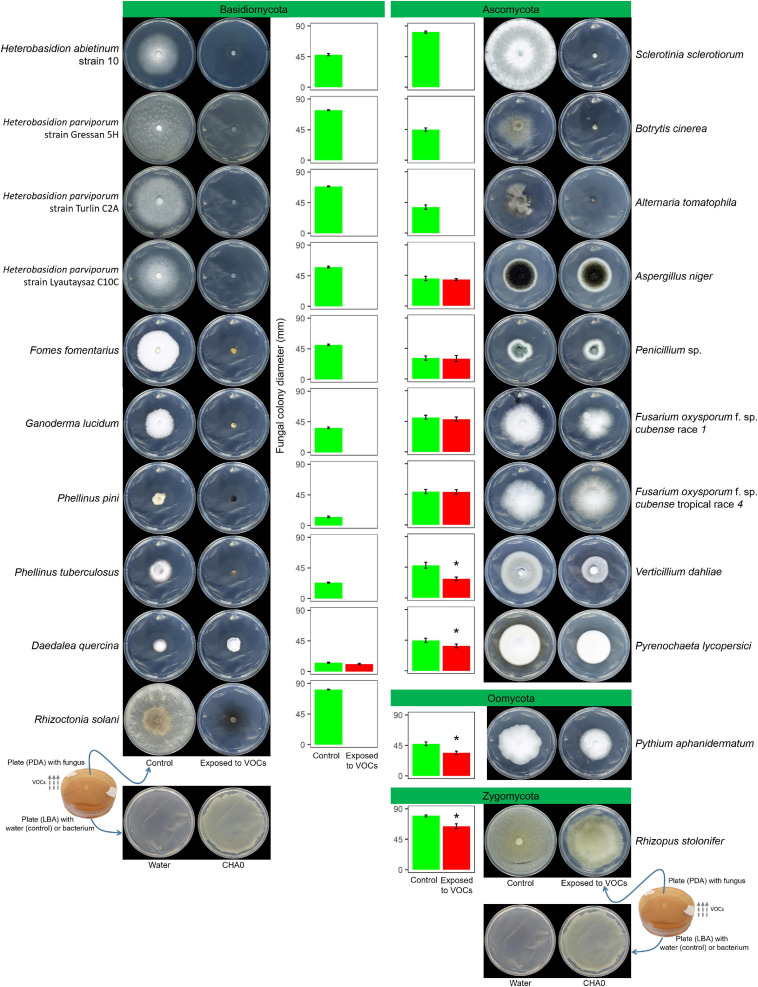
*Experiment A*. Effects of volatile organic compounds (VOCs) emitted by *Pseudomonas protegens* CHA0 against several plant pathogens in the overlapping plate assay. Photos were taken 7 days after the plates were joined and sealed, i.e., 7 days of exposure to CHA0’s VOCs. In the graph, error bars are the standard error of the mean of three replicates (plates). Bars with the asterisk are significantly different from the control according to the LSD test (*P* < 0.05).

In Experiment A, the VOCs emitted by *P. protegens* CHA0 in the OP assay inhibited completely the growth of seven Basidiomycete species, including *H. abietinum*, *H. parviporum* (three strains), *F. fomentarius*, *G. lucidum*, *P. pini*, *P. tuberculosus*, and *R. solani* (Figure 1). The growth inhibition was so sudden and potent that no mycelium emerged from the colonized agar plug placed in the agar plates. On the other hand, the growth of the Basidiomycete *D. quercina* was not affected; the colony exposed to VOCs grew very slowly but was not different from the control.

Among eight Ascomycete species, only three were highly sensitive to VOCs: *S. sclerotiorum*, *B. cinerea*, and *A. tomatophila* (100% inhibition). In contrast, *A. niger*, *Penicillium* sp., the race 1, and tropical race 4 of *F. oxysporum* f. sp. *cubense* were largely insensitive to VOCs (no significant growth reduction). The growth of *V. dahliae* and *P. lycopersici* was significantly reduced by 37 and 16%, respectively. The Oomycete *P. aphanidermatum* and the Zygomycete *R. stolonifer* were significantly inhibited by 25 and 18%, respectively.

### A Wide Range of Bacterial Species Inhibit *H. abietinum*

After finding that most Basidiomycetes were highly sensitive to CHA0’s VOCs, we investigated whether this trait was specific to *P. protegens* CHA0 or present in other bacteria. *Heterobasidion abietinum* strain 10 was used as a representative Basidiomycete for this and all the successive experiments. Therefore, in Experiment B, we reproduced the previous experiments using several strains of beneficial and plant pathogenic bacteria: *Pseudomonas* spp., *Bacillus* spp., *E. coli*, *P. carotovorum* subsp. *carotovorum*, *P. syringae* pv. *tomato*, and *A. tumefaciens*.

From Supplementary Figure 2, there is no doubt that all the tested bacteria prevented *H. abietinum* growth. Since taxonomically distant bacteria equally impeded *H. abietinum* growth, a very general mechanism was likely behind this phenomenon.

### Damages Caused by *P. protegens* CHA0’s VOCs to *H. abietinum* Are Severe and Rapid

**FIGURE 2:**
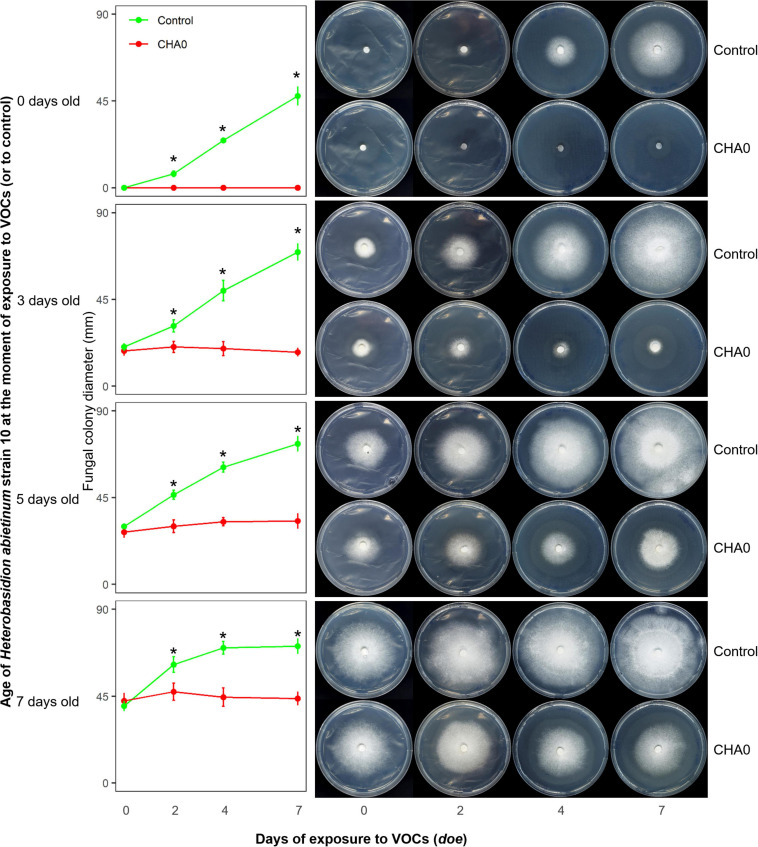
*Experiment C.* Effect of volatile organic compounds (VOCs) emitted by *Pseudomonas protegens* CHA0 on differently aged mycelia of *Heterobasidion abietinum* strain 10. The photos show colonies of *H. abietinum* strain 10 grown on potato dextrose agar (PDA) and exposed to VOCs emitted by CHA0 grown on Luria-Bertani agar (LBA). Halos around the colonies were due to diffusible substances secreted by the fungus and did not contain hyphae. In the graphs, error bars are the standard error of the mean of three replicates (plates). Asterisks indicate a significant difference between VOC-exposed colonies and the control according to the LSD test (*P* < 0.05). The growth of VOC-exposed colonies did not significantly differ over time (at 2, 4, and 7 days of exposure to VOCs, or *doe*, compared to control, i.e., 0 *doe*).

**FIGURE 3:**
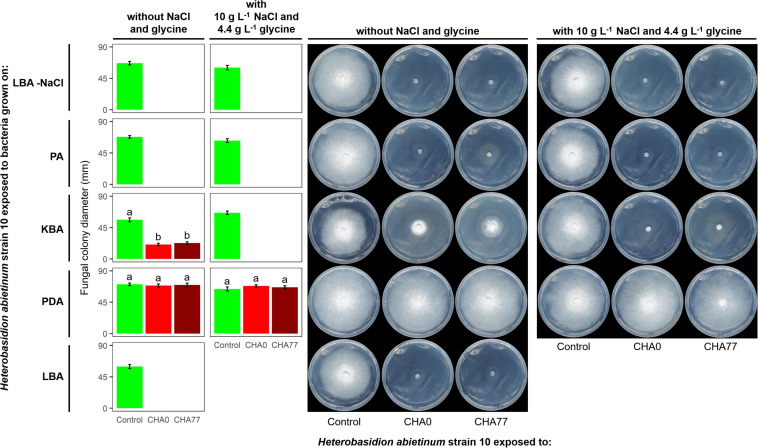
*Experiment D*. Effect of different agar media on the production of anti-Basidiomycete volatile organic compounds (VOCs) by *Pseudomonas protegens* strains CHA0 and CHA77. Photos were taken 10 days after the plates were joined and sealed, i.e., 10 days of exposure to *P. protegens*’ VOCs. The photos show colonies of *Heterobasidion abietinum* strain 10 grown on potato dextrose agar (PDA) and exposed to VOCs emitted by CHA0 or CHA77 grown on different media such as PDA, Luria-Bertani agar (LBA), King’s B agar (KBA), or peptone agar (PA), either with NaCl and glycine or not. Halos around the colonies were due to diffusible substances secreted by the fungus and did not contain hyphae. In the graph, error bars are the standard error of the mean of three replicates (plates). Bars with different letters are significantly different according to the LSD test (*P* < 0.05).

In Experiment C, we tested the hypotheses that the sensitivity to VOCs of the fungus-colonized agar plugs used in the OP assay was due to the very young age of hyphae inside it, and/or the wounds to hyphae generated during sampling from the source colony. Colonies of *H. abietinum* strain 10 of different ages were exposed to CHA0. This should demonstrate whether mature fungal colonies, e.g., 5 or 7 days old, resisted the damage caused by the CHA0’s VOCs more than young ones, e.g., colonized agar plug or a 3-day-old colony. In Figure 2, it is evident that the growth of *H. abietinum* strain 10 was stopped independently of its age. When plates were exposed to VOCs immediately after inoculation (0 days old, 6 mm colonized agar plug), *H. abietinum* colonies did not grow at all, while the control grew up to 53.6 mm in diameter after 7 days of exposure (*doe*) to VOCs. When plates were exposed 3 days after inoculation (3 days old *H. abietinum* colonies), control grew from 18.3 to 69.5 mm in diameter, while CHA0-exposed colonies measured on average 20.3 mm at 0 *doe* and 17.6 mm at 7 *doe*. A similar pattern occurred for 5-day- and 7-day-old colonies (Figure 3). Such a time course of the bacterium-fungus interaction suggested that the fungal growth was stopped suddenly at the moment of exposure to VOCs.

In Figure 2, especially the pictures taken at 7 *doe*, it can be seen that fungal colonies exposed to VOCs were surrounded by a halo. Observation under the optical microscope revealed that few, weak hyphae were present in that zone of agar (data not shown). Very likely, such hyphae in the agar were partially protected from the VOC damage, while the aerial mycelium was fully exposed and thus promptly destroyed.

To further evaluate the magnitude of fungal growth inhibition mediated by VOCs, an OP assay with an increasing number of CHA0 colonies was established. In Supplementary Experiment 1, very few CHA0 colonies were enough to inhibit *H. abietinum* growth (Supplementary Figure 3). One bacterial colony reduced the fungal growth by 26%. Such a fungal growth reduction reached 77 and 91% with two and five bacterial colonies, respectively. Nine or more colonies inhibited completely *H. abietinum*.

### Hydrogen Cyanide (HCN) Is Not Involved in the Basidiomycete Growth Inhibition

Hydrogen cyanide is one of the most common antifungal volatile produced by bacteria (Voisard et al., 1989). Therefore, we evaluated the effect of HCN against *H. abietinum* as the first mechanistic step of our study. An HCN-deficient mutant of CHA0, namely CHA77, and two compounds known to stimulate HCN production, such as NaCl and glycine (Castric, 1977; Blumer and Haas, 2000), were used to assess the participation of HCN in the fungal growth inhibition. As expected, Supplementary Figure 4 shows that HCN was produced only by CHA0 but it was not detected in CHA77. Interestingly, in Experiment D, no difference between CHA0 and CHA77 was observed on any media, except for KBA (Figure 3). In particular, both strains stopped the fungal growth when grown on LBA and PA, with or without NaCl and glycine. The fungus exposed to amended KBA was completely inhibited, while when exposed to non-amended KBA its growth was significantly reduced by 54–57%. No growth reduction occurred on the fungus exposed to PDA. These pieces of evidence, viz. no difference between CHA0 and CHA77 and between amended and non-amended media, indicated that HCN was not involved in the *H. abietinum* inhibition mediated by *P. protegens* CHA0’s VOCs. Indeed, if HCN was the main anti-Basidiomycete compound, no or significantly reduced fungal inhibition should occur with CHA77.

### VOCs Effective Against Basidiomycetes Are Produced by *P. protegens* CHA0 Only When Grown on Media Containing Digested Proteins

It has been known that nutrient availability can affect significantly the growth of microbes and thus the production of metabolites, including VOCs (Fernando et al., 2005; Blom et al., 2011). Indeed, based on the growth of *H. abietinum* strain 10, the previous Experiment D demonstrated a marked effect of the growth medium on the production of VOCs by *P. protegens* (Figure 3). Fungal growth was inhibited by 100% when *P. protegens* CHA0 was grown on LBA and PA, while it was reduced by 54–57% when the bacterium was grown on KBA (left panel of plates in Figure 3). No inhibition occurred using PDA. Therefore, all media containing digested proteins of variable origin stimulated the production of anti-*Heterobasidion* VOCs in CHA0.

It is noteworthy that *H. abietinum* growth rate in the control plates (i.e., exposed to plates without the bacteria) varied amongst the different media. At 10 dpi, The largest growth occurred when exposed to PDA, while the exposition to LBA and KBA induced a significant growth reduction of 14 and 19%, respectively (left panel of plates in Figure 3). Exposition to PA did not cause significant growth reduction compared to PDA.

The clear-cut difference between LBA and PDA in inducing effective VOCs was also demonstrated for bacteria other than CHA0 (Supplementary Figure 5), i.e., those used in Experiment B.

### Ammonia Plays a Major Role in the Inhibition of *H. abietinum* Mediated by *P. protegens* CHA0’s VOCs

Ammonia has been known to participate in the biocontrol mediated by beneficial bacteria (Tsao and Oster, 1981; Punja and Grogan, 1982; Punja et al., 1986; DePasquale and Montville, 1990; Tenuta and Lazarovits, 2002; Samapundo et al., 2007). Both CHA0 and CHA77, along with several other bacteria tested in this research, were proved to produce ammonia (Supplementary Figure 6).

**FIGURE 4:**
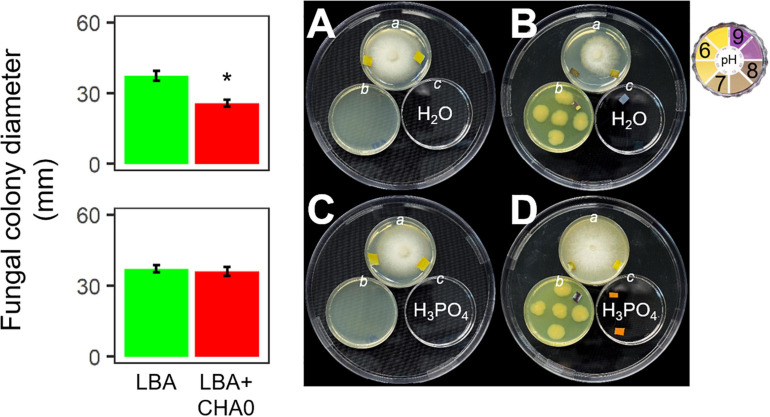
*Experiment E.* Involvement of ammonia in the growth inhibition of *Heterobasidion abietinum* strain 10 (plates in the top angle of the triangles) mediated by *Pseudomonas protegens* CHA0 (plates in the bottom left angles of the triangles). The 55 mm-plates labeled with *a* contained PDA and were inoculated with *H. abietinum* strain 10. The plates *b* contained LBA and were inoculated with CHA0 (150 mm-plates, **B,D**) or uninoculated **(A,C)**. The plates *c* contained water **(A,B)** or phosphoric acid **(C,D)**. The three 55 mm-plates were incubated without lid inside the 150 mm-plates which were sealed with Parafilm^®^ M to avoid the escape of volatiles emitted by CHA0. Pieces of litmus paper were placed on the agar surface at the photo capturing time to check its pH.

Experiment E was carried out to test the hypothesis that ammonia played a major role in the CHA0-*Heterobasidion* interaction. CHA0’s VOCs inhibited *H. abietinum* growth when both the bacterium and the fungus grew in an environment (150 mm-plate) with a plate (55 mm; *c*) containing sterile distilled water (control) (Figure 4B). Interestingly, the mycelium (plate *a*) grew better toward the water plate (*c)* than toward the bacterium plate (*b*). Also, with the aid of litmus paper, it was evident that the agar pH in the fungus plate (*a*) increased in the zone near the bacterium (*b*) more than in the zone near the water plate (*c*). In contrast, in the presence of phosphoric acid (*c*), the fungal growth inhibition and pH changes were abolished (Figure 4D). The fungus grew normally in the control plates (Figures 4A,C). These two pieces of evidence suggested that ammonia emitted by CHA0 (*b*) migrated in the fungal headspace (*a*) and exerted its toxicity. But in the presence of phosphoric acid (*c*), this compound continuously subtracted ammonia (to form ammonium phosphate salts) from the fungus headspace (*a*) (Ryden and McNeill, 1984) as it was produced by the bacterium (*b*), thus avoiding the toxic effect on the fungus (Figure 4B). Therefore, our hypothesis was confirmed.

**FIGURE 5:**
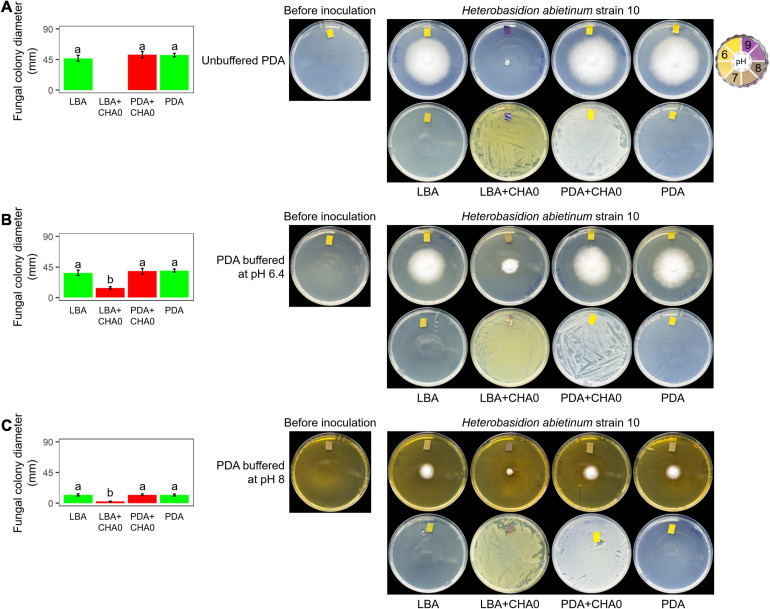
*Experiment F*. Alkalization of potato dextrose agar (PDA), unbuffered **(A)** or buffered at pH 6.4 **(B)** or 8 **(C)**, mediated by the volatiles of *Pseudomonas protegens* CHA0, and effect on the growth of *Heterobasidion abietinum* strain 10. Halos around the fungal colonies were due to diffusible substances secreted by the fungus and did not contain hyphae. In the graphs, error bars are the standard error of the mean of three replicates (plates). Within each graph, bars with different letters are significantly different according to the LSD test (*P* < 0.05).

Experiment F aimed at evaluating whether *H. abietinum* was affected directly by bacterial volatiles (including ammonia) or indirectly by the agar pH alkalization due to the volatiles. The volatiles emitted by CHA0 completely inhibited *H. abietinum* only with the bacterium grown on LBA (Figure 5A), as already shown in the previous Experiment D (Figure 3). Also, the volatiles emitted from CHA0 grown on LBA strongly alkalized (ca. pH 9) the media both with the bacterium and, at distance, with the fungus. On PDA buffered at pH 6.4, such alkalization was limited to ca. pH 7.5, and *H. abietinum* growth was strongly, but not completely, inhibited (60% reduction in diameter at 7 days post-inoculation) by CHA0’s volatiles (Figure 5B). On PDA buffered at pH 8, alkalization of the media reached ca. pH 8, and the fungus growth was reduced by 80% Figure 5C). The inhibition that occurred on buffered media, though to a diverse extent, indicated that volatiles impacted directly the fungal growth. It should be noted that *H. abietinum* growth was also reduced in control plates (without CHA0) containing either PDA buffered at pH 6.4 (23% reduction) and 8 (75% reduction), indicating that high pH disturbed the fungus. Collectively, it could be concluded that the *H. abietinum* was injured by CHA0’s volatiles both directly and indirectly (i.e., through alkalization of the medium).

### Effective VOC Candidates Were Identified by GC/MS

By analyzing the differential VOCs emitted by CHA0 grown on LBA or PDA, and those emitted by the uninoculated media, we were able to identify volatile candidates potentially involved in the *H. abietinum* growth inhibition, in addition to the ammonia mentioned above (Experiment G). The PCA revealed that VOCs from CHA0 grown on LBA clustered separately from both those of CHA0 grown on PDA and uninoculated medium controls (LBA and PDA) (Supplementary Figure 7A). This correlated with the toxic effect of the CHA0’s volatiles observed only when the bacterium was grown on LBA. The difference in VOCs emitted by PDA with or without CHA0 was not very large, very likely as a consequence of a non-optimal growth on this medium. Hierarchical clustering (heatmap) showed the presence of mass features (mz/rt) specifically abundant (brownish shades) under all four different conditions (Supplementary Figure 7B). ANOVA and *post hoc* analysis revealed significantly (*P* < 0.05) different mass features present in all four conditions (red dots in Supplementary Figure 7C). Eleven compounds, including three unknown, were found differentially emitted (Table 1). 1,3-Diphenylpropane was the substance identified with the highest probability (94.59%), followed by dimethyl trisulfide (90.96%). The probability of identification of the other substances ranged between 62.28 and 82.31%. Three unknown substances were identified with lower probability, i.e., 41.09, 33.24, and 5.84%.

### Ammonia, Dimethyl Trisulfide, and Other Volatiles Are Effective *in vitro* Against *H. abietinum*

In Experiment H, seven synthetic VOCs were tested *in vitro* against *H. abietinum* strain 10 to evaluate their toxicity. These compounds were those identified and named by the GC/MS analysis, except acetonanil, which was commercially unavailable, and the three compounds that remained unnamed in our analysis (Experiment G; Table 1). All the compounds except butyldiglycol completely inhibited the fungus when tested as a pure compound, but they displayed variable effectiveness when diluted at several rates (Table 2). Dimethyl trisulfide was the most toxic compound, with an EC_50_ of 0.08 μL and a MIC of 1 μL. This meant that *H. abietinum* did not grow in a Petri plate if 1 μL dimethyl trisulfide was placed on the lid and volatilized in the headspace of the fungus. The fungus growth was reduced by 50% compared to control if 0.08 μL (amount estimated by interpolation) was placed on the plate lid. EC_50_ values ranging between 0.86 and 11.03 μL and MIC values of 2–50 μL were estimated for 2-ethylhexanol, ammonium hydroxide, phenol, acetophenone, and dimethyl disulfide (Table 2). 1,3-Diphenylpropane and 3-phenylpropiophenone were the least toxic compounds with EC_50_ of 59.33 and 67.19 μL, respectively, and MIC higher than 100 μL. Finally, butyldiglycol did not exhibit toxicity against the fungus. Unlike ammonium hydroxide (Figure 4), none of these compounds increased the pH level of agar media at distance, as ascertained with litmus paper (data not shown). This experiment confirmed the high toxicity of ammonia and classified dimethyl trisulfide as the most toxic compound amongst those identified in the GC/MS analysis.

**TABLE 2:** *Experiment H. In vitro* toxicity of synthetic volatile compounds against *Heterobasidion abietinum*.

Volatile compounds	Effective concentration 50% (EC_50_)		Minimum inhibitory concentration (MIC)	
		
	μL*^*a*^*	Molarity*^*b*^*	μL*^*a*^*	Molarity*^*b*^*
Dimethyl trisulfide	0.08 ± 0.002 a	0.02 ± 0.002 a	1	0.2
2-Ethylhexanol	0.86 ± 0.01 b	0.33 ± 0.01 b	2	0.77
Ammonium hydroxide	1.65 ± 0.03 a	1.18 ± 0.02 a	3	2.14
Phenol	3.76 ± 0.21 d	2.00 ± 0.11 d	10	5.31
Acetophenone	5.81 ± 0.20 e	1.21 ± 0.04 c	20	4.16
Dimethyl disulfide	11.03 ± 0.28 f	2.93 ± 0.07 e	50	13.27
1,3-Diphenylpropane	59.33 ± 7.48 g	7.56 ± 2.23 f	>100	>12.74
3-Phenylpropiophenone	67.19 ± 0.34 g	15.98 ± 0.08 g	>100	>23.78
Butyldiglycol	n.t.	n.t.	n.t.	n.t.

*^a^ Microliters poured on the lid of 90 mm-Petri plates placed upside down and containing PDA inoculated with Heterobasidion abietinum.*

*^b^ Molarity of the compound relative to the air space above the agar surface in the Petri plate, which was estimated to be 40 mL in the experiment.*

*Data are presented as the average of three replicates ± standard error. Within each column, means with different letters are significantly different according to the LSD test (*P* < 0.05). Amounts of compounds smaller than 10 μL were diluted in dimethyl sulfoxide to allow their handling. n.t., no toxicity.*

Then, in Experiment I, ammonia and dimethyl trisulfide were tested against some fungal strains already used in Experiment A to ascertain whether the sensitive/resistant responses could be reproduced using synthetic compounds instead of the bacterium-emitted volatiles. In Experiment A, almost all tested Basidiomycete strains were sensitive to CHA0’s VOCs while, in the Ascomycota phylum, some strains were sensitive and others were resistant. Experiment I confirmed some results obtained from Experiment A. *Heterobasidion abietinum*, *S. sclerotiorum*, and *B. cinerea* were sensitive in Experiment A and showed the lowest EC_50_ (ranging from 1.55 to 1.65 μL) and MIC (3–6 μL) values for ammonium hydroxide in Experiment I (Table 3). *Heterobasidion abietinum* also showed the lowest EC_50_ (0.08 μL) and MIC (1 μL) values for dimethyl trisulfide. *Fusarium oxysporum* f. sp. *cubense* (Ascomycete) was resistant in Experiment A and showed the highest EC_50_ and MIC values for both ammonium hydroxide and dimethyl trisulfide. On the other hand, results from these two experiments were controversial for *R. solani* (Basidiomycete) and *V. dahliae* (Ascomycete). The two fungi were sensitive and resistant to CHA0’s VOCs, respectively, but did not show significantly different EC_50_ and MIC values for both ammonium hydroxide and dimethyl trisulfide. *Sclerotinia sclerotiorum* and *B. cinerea* (Ascomycetes) were sensitive and showed EC_50_ and MIC values intermediate between *H. abietinum* (sensitive) and *F. oxysporum* f. sp. *cubense* (resistant). The discrepancy between the two experiments for some fungi might imply that the toxicity was due to yet unidentified compounds or to the mixture of volatiles rather than to single compounds.

**TABLE 3:** *Experiment I. In vitro* toxicity of ammonium hydroxide and dimethyl trisulfide against *Heterobasidion abietinum* and other fungi.

Synthetic compounds and fungi	Effective concentration 50% (EC_50_)		Minimum inhibitory concentration (MIC)		Phylum	Response to CHA0’s VOCs*^*c*^*
		
	μL*^*a*^*	Molarity*^*b*^*	μL*^*a*^*	Molarity*^*b*^*		
**Ammonium hydroxide**						
*H. abietinum^*d*^*	1.65 ± 0.03 a	1.18 ± 0.02 a	3	2.14	Basidiomycete	S
*R. solani*	4.45 ± 0.06 b	3.17 ± 0.04 b	6	4.28	Basidiomycete	S
*S. sclerotiorum*	1.59 ± 0.06 a	1.13 ± 0.04 a	3	2.14	Ascomycete	S
*B. cinerea*	1.55 ± 0.03 a	1.11 ± 0.02 a	3	2.14	Ascomycete	S
*V. dahliae*	4.43 ± 0.13 b	3.16 ± 0.09 b	6	4.28	Ascomycete	R
*F. oxysporum* f. sp. *cubense*	8.65 ± 0.53 c	6.17 ± 0.38 c	15	10.7	Ascomycete	R
**Dimethyl trisulfide**						
*H. abietinum^*d*^*	0.08 ± 0.002 a	0.02 ± 0.002 a	1	0.2	Basidiomycete	S
*R. solani*	0.53 ± 0.02 b	0.1 ± 0.003 b	1	0.2	Basidiomycete	S
*S. sclerotiorum*	0.53 ± 0.01 b	0.1 ± 0.003 b	1	0.2	Ascomycete	S
*B. cinerea*	0.51 ± 0.01 b	0.1 ± 0.002 b	1	0.2	Ascomycete	S
*V. dahliae*	0.48 ± 0.02 b	0.1 ± 0.002 b	1	0.2	Ascomycete	R
*F. oxysporum* f. sp. *cubense*	4.36 ± 0.12 c	0.86 ± 0.02 c	10	1.98	Ascomycete	R

*^a^ Microliters poured on the lid of 90 mm-Petri plates placed upside down.*

*^b^ Molarity of the compound relative to the air space above the agar surface in the Petri plate, which was estimated to be 40 mL in the experiment.*

*^c^ Response to volatile organic compounds emitted by Pseudomonas protegens CHA0 as observed in Experiment A: S, sensitive; R, resistant.*

*^d^ Data for H. abietinum were obtained from Experiment H and reported here for comparison.*

*Data are presented as the average of three replicates ± standard error. Within each column, means with different letters are significantly different according to the LSD test (P < 0.05). Amounts of compounds smaller than 10 μL were diluted in dimethyl sulfoxide to allow their handling. n.t., no toxicity.*

### *H. abietinum* Hyphae Are Injured Very Early After Exposure to *P. protegens* CHA0’s VOCs

**FIGURE 6:**
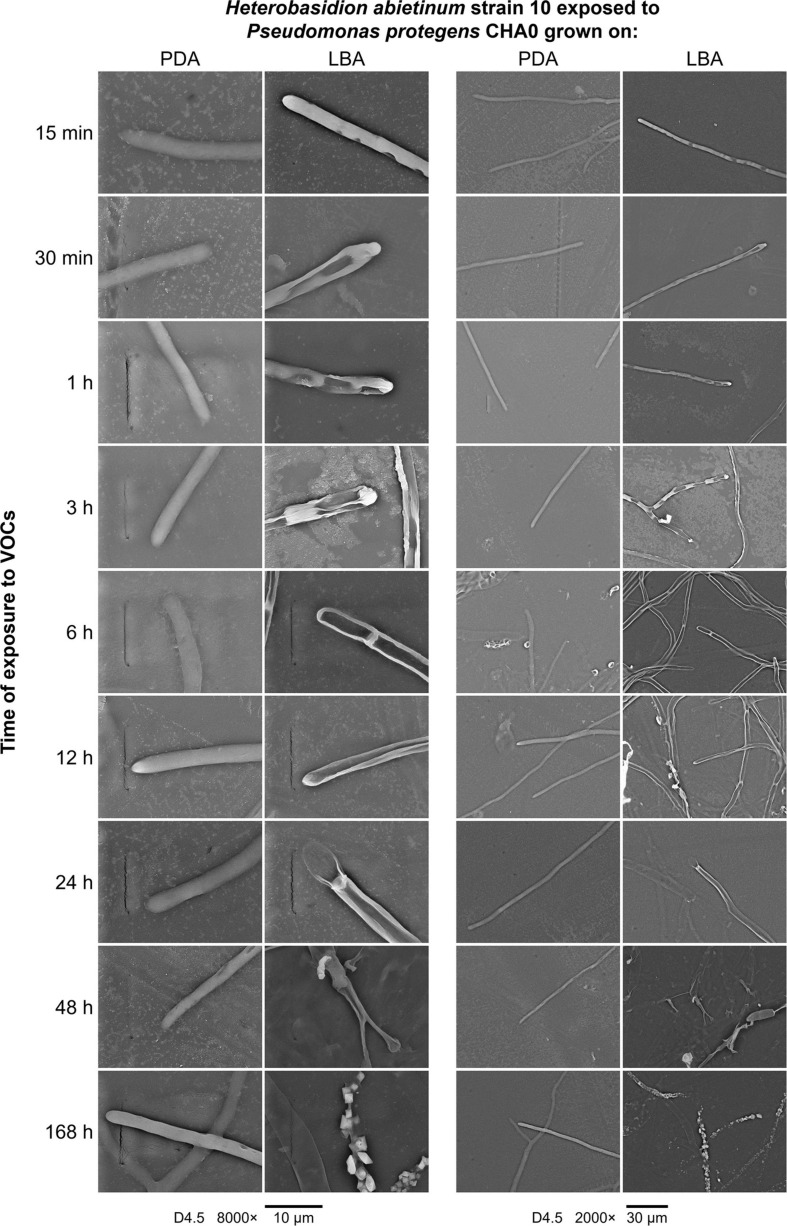
*Experiment J.* Observations at the scanning electron microscope (SEM) of the effect of volatile organic compounds (VOCs) emitted by *Pseudomonas protegens* CHA0 when grown on Luria-Bertani agar (LBA) rather than on potato dextrose agar (PDA) against *Heterobasidion abietinum* strain 10. The photos show hyphal tips of *H. abietinum* strain 10 damaged by the VOCs emitted from CHA0 grown on LBA.

A series of SEM observations were used to describe the time course of the injuries caused by VOCs on *H. abietinum* hyphae (Experiment J). Fungal hyphae exposed to CHA0 grown on LBA were compared to those exposed to the same bacterium grown on PDA (i.e., LBA vs. PDA). The earliest time point of observation was 15 min after exposure to VOCs, and the latest one was 168 h later. During the entire experiment, control hyphae were observed to be turgid and with intact cell wall, indicating their good health and the absence of deleterious effects due to sample preparation and extreme conditions (freezing and vacuum) during SEM observations (PDA panels in Figure 6). At 15 min after exposure to VOCs, with 8000× magnification, some sunken lesions were observed on the cell wall of the VOC-exposed hyphae, immediately behind the tip (LBA panels in Figure 6). Such early injury was in line with our perception that fungal growth was stopped suddenly upon exposure to VOCs, as argued for the experiments with differently aged fungal colonies (Figure 2). At 30 min after exposure, such sunken lesions were more severe and extended, so that the hyphae shrunk in most of their length. In the successive time points, the hyphal cells delimited by septa appeared shrunk and emptied. At 48 and 168 h post-exposure, the mycelium was destroyed.

### Cell Membrane and Cytoplasmic Matrix of *H. abietinum* Are Injured by *P. protegens* CHA0’s VOCs

TEM observations of ultra-thin sections revealed severe alterations of *H. abietinum* hyphae due to the exposure to VOCs emitted by CHA0 grown on LBA (Experiment K).

**FIGURE 7:**
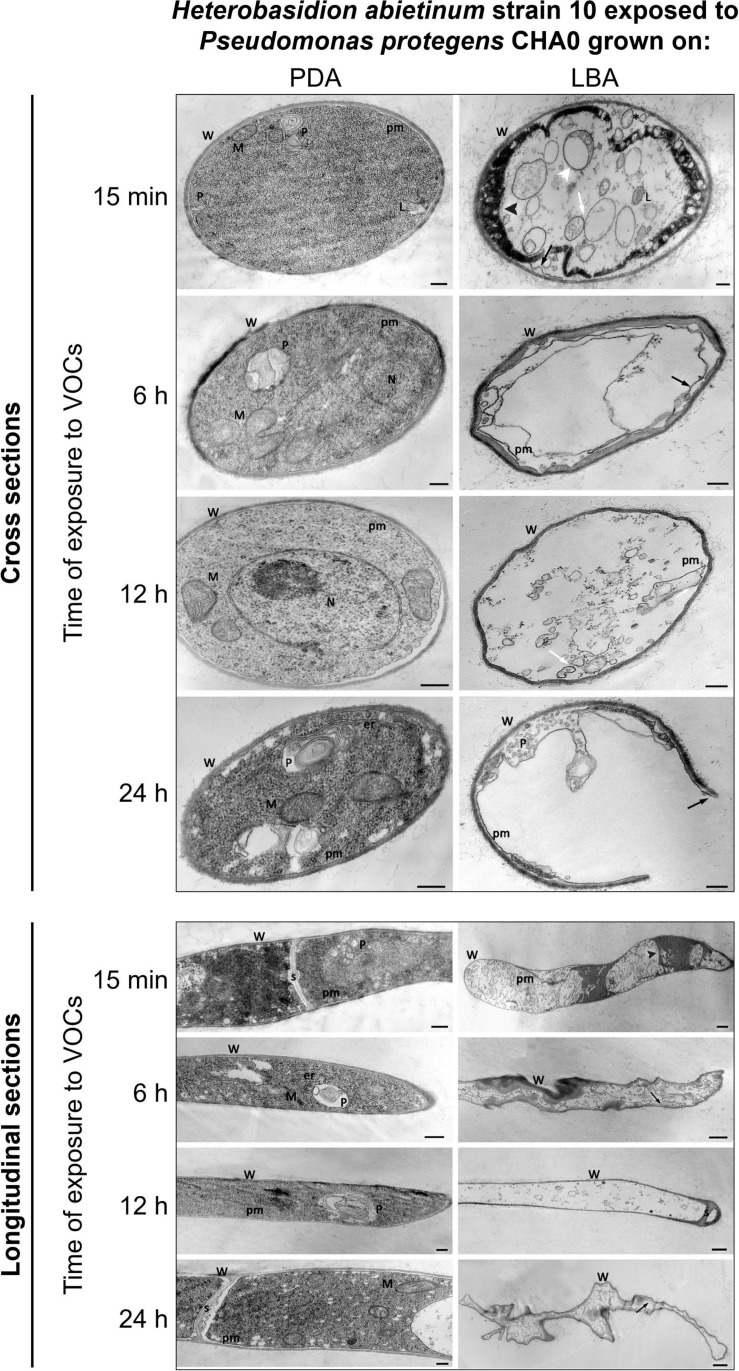
*Experiment K.* Transmission electron microscope (TEM) observations. Hyphae of *Heterobasidion abietinum* strain 10 exposed to volatile organic compounds (VOCs) emitted by *Pseudomonas protegens* CHA0 grown on Luria-Bertani agar (LBA) compared to the control, i.e., fungal hyphae exposed to VOCs of the same bacterium grown on potato dextrose agar (PDA). Hyphae were sampled from an overlapping plate assay at 15 min, 6, 12, and 24 h of exposure to VOCs. Black arrow: plasma membrane detachment and rupture; white arrowhead: mitochondria vesiculation a; white arrow: pseudo-vacuoles; black arrowhead: denser cytoplasm. Asterisk: lomasome/plasmalemmasome residues; W, cell wall; M, mitochondrion; N, nucleus; L, lomasome; P, plasmalemmasome; er, endoplasmic reticulum; pm, plasma membrane; s, septum. Bars: 250 nm for the panels of longitudinal sections/PDA and cross-sections, and 500 nm for the panel of longitudinal sections/LBA.

As soon as 15 min of exposure (*moe*) to VOCs, ruptures of the plasma membrane and its detachment from the cell wall occurred (black arrow in Figure 7). Such breaks impacted directly the inner structure of the cell as indicated by the presence of lomasome/plasmalemmasome residues (asterisk), dense cytoplasm areas (black arrowhead), mitochondria vesiculation (white arrowhead), and pseudo-vacuoles (white arrow), which are all signs of cell deterioration. Even at this early stage, abnormal cell vacuolization, cytoplasmic matrix deterioration, loss of ribosomes, mitochondria vesiculation, and disintegration of other organelles occurred.

At 6 *hoe*, cross-sections revealed an angular-shaped cell profile indicating that hyphae became shrunk, as also manifest in the longitudinal sections. Also, hyphae already became partially empty, very likely due to cytoplasm leakage through the plasma membrane ruptures that were still evident.

At 12 *hoe*, the plasma membrane further degenerated and only a few residues could be observed. Nevertheless, similarly to the previous time point, the cell wall was deformed but not broken; septa between hyphal cells were still intact. At 24 *hoe*, ruptures occurred in the cell wall, and hyphae became severely degenerated.

### *P. protegens* CHA0’s VOCs Compromise Protein Synthesis and Induce Defense Mechanisms in *H. abietinum*

Severe and rapid injuries caused by LBA grown-CHA0’s VOCs on *H. abietinum* mycelium were observed. Using RNA-Seq, we aimed at understating the molecular basis of such injuries. Hence, RNA was extracted and sequenced from hyphae exposed for 12 h to VOCs emitted by CHA0 grown on LBA and compared to the transcriptome of hyphae exposed to the bacterium grown on PDA (viz. LBA vs. PDA), where no injuries were observed (Experiment L).

Volatile organic compounds induced significant transcriptome changes in *H. abietinum* strain 10, as evidenced by the PCA (Supplementary Figure 8A). Among 13,384 genes in the transcriptome, 471 were found differentially expressed (FDR < 0.05) in the analyzed samples, including 246 up- and 225 down-regulated genes (Supplementary Figure 8B and Supplementary Table 2). Although already available, we performed a new annotation process of the genes and successfully annotated almost all genes (12,996) with GO, 10,762 genes with InterPro domains, and 1,099 with enzyme Commission numbers (EC). These data, reported in Supplementary Table 2, represent updated and improved annotations of genes compared to those originally reported in the JGI Genome Portal for the reference strain *H. annosum* TC 32-1 v. 2.0.

The enrichment analysis of GO revealed that 335 (180 up- and 155 down-regulated) out of 471 DEGs belonged to enriched GOs (FDR < 0.05). Similarly, only 383 genes, including 30 up- and 13 down-regulated DEGs, were linked to KEGG pathways and used for the enrichment analysis. The GO enrichment analysis results are reported in Supplementary Table 3: 133 GO terms were enriched in the biological process category, 61 in the molecular function, and 13 in the cellular component. All these enriched GO terms were used to construct hierarchical graphs. For better readability, only representative branch nodes of those graphs are shown in Supplementary Figures 9, 10.

**FIGURE 8:**
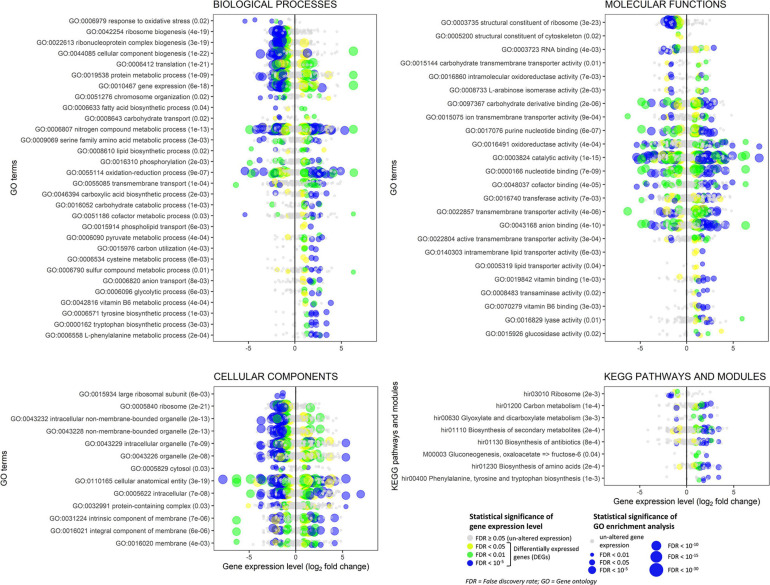
*Experiment L.* Enrichment analysis of the gene ontology terms and the KEGG pathways and modules for the RNA-Seq experiment where *Heterobasidion abietinum* strain 10 was exposed to the volatile organic compounds (VOCs) emitted by *Pseudomonas protegens* CHA0 grown on Luria-Bertani agar (LBA) versus potato dextrose agar (PDA). For the biological processes and molecular functions, only 30 out of 133 and 24 out of 61 GO terms, respectively, are showed for better readability; these GO terms were representative nodes of the branches in the hierarchical graphs induced by all the enriched terms. Dots are gene expression means of three biological replicates (log_2_ fold change compared to control). In parentheses next to the term description, the significance of enrichment analysis is reported (FDR).

Among the most significantly enriched GOs (FDR ranging from 10^–23^ to 10^–19^), several terms related to ribosomes occurred in the biological process (e.g., GO:0042254 ribosome biogenesis), molecular function (e.g., GO:0003735 structural constituent of ribosome), and cellular component categories (e.g., GO:0005840 ribosome) (Figure 8 and Supplementary Figures 9, 13). Forty-seven DEGs were associated with these GOs and 45 of them were down-regulated (Figure 8 and Supplementary Figure 11). Structural constituents of the cytoskeleton such as *alpha-tubulin* genes (geneIDs 62388, 146577, and 411958) were also down-regulated (log_2_FC from −0.95 to −0.61). The *mitochondrial outer membrane translocase receptor subunit TOM70* (geneID 156777) was one of the few genes up-regulated (log_2_FC = 1.4) in the GOs related to ribosomes. Most genes involved in the translation were down-regulated, though some tRNA ligases such as those of isoleucine (geneID 319333), alanine (geneID 476425), and aspartate (geneID 437829) were induced. These up-regulated tRNA ligases, together with few other up-regulated genes such as *histone acetyltransferase* (geneID 54722, log_2_FC = 3.62), *mitochondrial carrier protein* (geneID 154123, log_2_FC = 2.09), and *prenyltransferase* (geneID 142593, log_2_FC = 1.99), were also associated to the protein metabolic process and gene expression where, however, the most part of genes was down-regulated; in these GOs, several mitogen-activated protein kinases were variably regulated with diminished (log_2_FC < −2.1, e.g., geneIDs 64560, 157315, 246629, 324951, and 454430) or augmented (log_2_FC > 1, e.g., geneIDs 45774, 62517, 239605, and 389878) expression levels compared to control.

In contrast to such detrimental effects on ribosome, translation, and gene expression, the biosynthesis of some amino acids, including tyrosine, tryptophan, and L-phenylalanine was overall induced. It was due to enzymes such as the 3-deoxy-7-phosphoheptulonate synthase (geneID 318732, log_2_FC = 3.4), aldehyde dehydrogenase [NAD(P)(+)] (geneID 124446, log_2_FC = 2.6), shikimate dehydrogenase (geneID 331603, log_2_FC = 2.3), aspartate transaminase (geneIDs 414741 and 437828, log_2_FC = 2.2), chorismate synthase (geneID 309507, log_2_FC = 1.8), and anthranilate synthase (geneID 154513, log_2_FC = 1.3). These genes act at various levels in the biosynthesis pathways of these amino acids (Supplementary Figure 12), and they also participate in the metabolic processes of cysteine (GO:0006534), sulfur compounds (GO:0006790), and vitamin B6 (GO:0042816). Several genes with aldehyde dehydrogenase [NAD(P)(+)] domain also functions in the glycolytic process (GO:0006096), pyruvate metabolic process (GO:0006090), oxidoreductase activity (GO:0016491), and oxidation-reduction process (GO:0055114). The seven DEGs associated with the glycolytic process were all up-regulated (log_2_FC ranging from 1 to 2.6). They were the *aldehyde dehydrogenase [NAD(P)(+)]* (geneID 124446), *acyl-CoA synthetase* (geneID 52436), *NAD-malate dehydrogenase* (geneID 146994), *6-phosphofructokinase* (geneID 474195), *fructose-bisphosphate aldolase* (geneID 410210), *glyceraldehyde 3-phosphate dehydrogenase* (geneID 419475), and *fructose-bisphosphatase* (geneID 414892). Peroxidases involved in the response to oxidative stress were strongly down-regulated (log_2_FC from −5.3 to −1.6, e.g., geneIDs 61259, 155921, 181069, 330082, and 408750). In the oxidation-reduction process (GO:0055114), three cytochrome P450 monooxygenases, viz. 17, 57 and 70 (geneIDs 442518, 68311, and 67634, respectively) were down-regulated (log_2_FC from −2.4 to −2.1) but the *cytochrome P450 monooxygenase 2* (geneID 147966) was up-regulated (log_2_FC = 4.9). Also, a number of dehydrogenases (geneIDs 37439, 108042, 124446, 146994, 155866, 331603, 388181, 411149, 419475, and 442100) and a *ferric reductase* (geneID 39263) were up-regulated (log_2_FC from 1 to 4.2).

The transmembrane transport (GO:0055085) was significantly altered. Several sugar transporters were up- (e.g., geneIDs 35934, 106254, and 148408; log_2_FC > 1.3) or down-regulated (e.g., geneIDs 62185, 106809, and 148438; log_2_FC < −1.6), as well as amino acid/peptide transporters which were up- (e.g., geneIDs 56213, 147314, and 153952; log_2_FC > 1.9) or down-regulated (geneID 61227; log_2_FC = −1.5). Contrasting expression levels also occurred for two aquaporins, i.e., geneIDs 60386 (log_2_FC = 3.2) and 51894 (log_2_FC = −2.2), two ion transporters, i.e., *ZIP-like iron-zinc transporter* (geneID 64133, log_2_FC = −6.4) and *Na+/H*+ *antiporter* (geneID 51284, log_2_FC = 1.8), and three P-type ATPases involved in the phospholipid transport (geneIDs 60087, 124278, 157655; log_2_FC from 0.9 to 1.7). The expression levels of seven genes annotated as ammonium transporters (geneIDs 66540, 99999, 104475, 306569, 331858, 407150, and 473428) were not significantly altered.

Several aforementioned genes are also involved in the biosynthesis of secondary metabolites and antibiotics, with most of them that were up-regulated: *glyceraldehyde-3-phosphate dehydrogenase* (geneID 419475), *fructose-bisphosphate aldolase* (geneID 410210), *6-phosphofructokinase* (geneID 474195), *anthranilate synthase* (geneID 154513), *chorismate synthase* (geneID 309507), *aspartate transaminase* (geneID 437828), *malate dehydrogenase* (geneID 146994), *shikimate dehydrogenase* (geneID 331603), *aldehyde dehydrogenase* [NAD(P)(+)] (geneID 124446), and *3-deoxy-7-phosphoheptulonate synthase* (geneID 318732).

The chromosome organization (GO:0051276) was affected because the expression of numerous genes was changed by log_2_FC ranging from −3.7 to 3.6, with peptidases (e.g., geneID 417918) and histone acetyltransferases among the up-regulated genes (e.g., geneIDs 54722 and 446283) and phosphatases among the down-regulated ones (e.g., geneIDs 154589 and 165527). Genes in this GO together with the constituents of the cytoskeleton (GO:0005200) might be involved in the cell division and development and, thus, in the growth inhibition of *H. abietinum* strain 10.

## Discussion

Seven out of eight tested Basidiomycete species were completely inhibited by CHA0’s VOCs. Such extensive toxicity was not found in the Ascomycota phylum, where some isolates were sensitive and others resistant (Figure 1). Our results on Oomycota and Zygomycota were not conclusive because only one isolate each was tested. Intriguingly, like CHA0, several phylogenetically distant bacteria inhibited the growth of *H. abietinum*, used in this research as a Basidiomycete model species (Supplementary Figure 2). These bacteria included 10 beneficial or pathogenic strains belonging to *Pseudomonas* spp., *Bacillus* spp., *E. coli*, *P. carotovorum* subsp. *carotovorum*, *P. syringae* pv. *tomato*, and *A. tumefaciens*. Therefore, a very general mechanism could be hypothesized for the growth inhibition of Basidiomycetes mediated by bacterial VOCs. The cell wall is the first physical barrier that VOCs encounter when they impact the fungal mycelium. Whether a difference in the cell wall composition among Basidiomycetes and other phyla could explain the different responses to bacterial VOCs would merit to be investigated. Variable sensitivity to enzymatic digestions has been ascribed to a diverse composition of the cell wall, even within the same phylum, that was Basidiomycota in early research conducted by Ballesta and Alexander (1972).

Hydrogen cyanide is one of the most common secondary metabolites involved in the biocontrol of plant pathogens (Voisard et al., 1989). It was unlikely that all bacteria used in our experiments produced HCN. Indeed, the fact that CHA77, an HCN-deficient mutant of CHA0, inhibited *H. abietinum* like CHA0 demonstrated that HCN did not participate in the phenomenon (Figure 3).

We further discovered that digested proteins were nutrients necessary for the production of bacterial VOCs effective against *H. abietinum*. The modulation of VOC blends by nutrient availability has been experienced by other researchers (Blom et al., 2011), and media richer in carbon and nitrogen sources or soils rich in organic matter have been associated with a higher VOC production by bacteria (Fernando et al., 2005). We found an intriguing scenario: *H. abietinum* was completely inhibited when exposed to bacteria grown on media containing peptone or tryptone, while the fungal growth was unaffected when exposed to bacteria grown on media without these compounds (Figure 3 and Supplementary Figure 5). Which VOCs were induced by the digested proteins?

It has been known that biogenic ammonia is a by-product of amino acid conversions like those of aspartate (Bernier et al., 2011) and glycine (Avalos et al., 2020). Ammonia exerts multiple functions in the cross-talk between bacteria and other organisms, spanning from toxicity for plants (Kai et al., 2010) to increased (Bernier et al., 2011) or reduced antibiotic resistance in bacteria (Avalos et al., 2020; Zhang et al., 2020). In our experiments, *H. abietinum* was sensitive to ammonia vapors that arose from ammonium hydroxide (NH_4_OH) solutions. Such vapors also increased agar pH at distance (Figure 4), a phenomenon also experienced in other studies (Letoffe et al., 2014; Jones et al., 2017; Avalos et al., 2020). Similarly, volatiles emitted by bacteria grown on digested proteins inhibited the fungus and alkalized the agar substrate (Figure 5). On the contrary, volatiles emitted by bacteria grown on media without digested proteins neither inhibited the fungus nor alkalized the medium. The ammonia production by bacteria grown on digested proteins was ascertained using Nessler’s reagent; they did not produce ammonia in the absence of digested proteins (Supplementary Figure 6). In our experiments, only ammonia was able to change the pH of agar media at distance, while the other VOCs identified in this work, tested as synthetic compounds, had not this capability. Therefore, we hypothesized that ammonia was amongst the main molecules responsible for *H. abietinum* inhibition and originated from the utilization of digested proteins by bacteria. This finding was particularly important for two reasons: (1) virtually all bacteria can utilize digested proteins and thus generate ammonia emissions, and (2) all tested Basidiomycetes were highly sensitive to volatiles emitted by bacteria grown on digested proteins.

Implications of this discovery in the control of Basidiomycete-incited diseases are multiple. Remarkably, it has long been known that urea is an effective control means against *Heterobasidion* species in forest environments populated especially with Norway spruce (*Picea abies*) (Brandtberg et al., 1996; Nicolotti and Gonthier, 2005; Thor and Stenlid, 2005; Oliva et al., 2008) or also with European larch (*Larix decidua*), silver fir (*Abies alba*), Scots pine (*Pinus sylvestris*) and other tree species (Pratt et al., 1998; Lehtijärvi et al., 2011; Wang et al., 2012; Gonthier and Thor, 2013). For example, in recent research conducted in the western Italian Alps, treatments with a 30% urea aqueous solution significantly reduced the percentage of infected Norway spruce stands from about 30% to 5%, and those of European larch from 12% to 5%, as recorded 2 years after treatment (Gonthier, 2019). In Sweden, urea has provided 92–94% and 95.5–99.8% control of *Heterobasidion* spp. infections in pre-commercial thinnings and final fellings, respectively (Blomquist et al., 2020). Urea is converted by ureases into ammonia and carbamic acid, which then reacts with water to form carbonic acid and ammonia. Urea treatment has been characterized by a strong selectivity against non-target fungi: isolation of fungi from freshly cut (7-week-old) stumps of Norway spruce treated with urea revealed that Basidiomycetes were almost eliminated while Ascomycetes predominated (Vasiliauskas et al., 2004). This is a fascinating connection with our experiments, where Basidiomycetes were all sensitive to bacterial volatiles and ammonia while both resistant and sensitive strains were found amongst Ascomycetes. Thus, there is a large body of evidence that ammonia, either synthetic or generated by bacteria, can drastically hamper the growth of Basidiomycetes.

Ammonia has been also proved toxic to Oomycetes (Tsao and Oster, 1981), soil-borne microsclerotia-producing fungi such as *Sclerotium rolfsii* and *V. dahliae* (Punja and Grogan, 1982; Punja et al., 1986; Tenuta and Lazarovits, 2002), and mycotoxigenic Ascomycetes belonging to the genera *Aspergillus*, *Penicillium* and *Fusarium* (DePasquale and Montville, 1990; Samapundo et al., 2007). Ammonia contributes to increasing pH, but elevated extracellular pH alone has not been found responsible for the antifungal activity in *P. griseofulvum* and *F. graminearum*. On the other hand, free ammonia, whose amount is however dependent on the pH, was associated with antifungal activity in these fungi (DePasquale and Montville, 1990). It has been known that many, if not all, bio-membranes are highly permeable to free ammonia (Macmillan, 1956; Desai and Modi, 1977; reviewed by Kleiner, 1981; Löffler et al., 1986) and that ammonium transporters (Amt) are ubiquitous membrane proteins found in all domains of life (Wirén and Merrick, 2004). In fungi, Amt proteins scavenge and recapture ammonium lost from cells across the membrane and act as ammonium sensors to mycelium growth (Wirén and Merrick, 2004). In our RNA-Seq experiment, several transmembrane transporters (of sugars, peptides, phospholipid, and ions) were differentially expressed but seven ammonium transporters were not affected.

Very likely, ammonia was not the sole molecule responsible for *H. abietinum* inhibition. By comparing the volatilomes of CHA0 grown on media containing (LBA) or not (PDA) digested proteins, several compounds were identified as specifically produced on LBA (Table 1). Among them, for the first time in a bacterial volatile blend, we identified 1,3-diphenylpropane, for which antibiotic activity has not been documented so far. 1,3-diphenylpropane is the skeleton of the flavonoids which are widely distributed in the plant kingdom (e.g., flower pigments). On the other hand, it could even be a contaminant deriving from the plastic (polystyrene) of Petri dishes because it is a styrene dimer. Therefore, its occurrence in a bacterial volatilome would merit further studies. Like in our experiments with CHA0, dimethyl disulfide, dimethyl trisulfide, and acetophenone have been detected in the volatile blends of the phyllospheric and rhizospheric *Pseudomonas* spp. with an anti-*Phytophthora* potential (De Vrieze et al., 2015). In this work, sulfur-containing VOCs (e.g., dimethyl trisulfide) and simple ketones were considered amongst the most important inhibitors of *Phytophthora infestans*. The toxicity of dimethyl trisulfide has been also proved against *Colletotrichum gloeosporioides* (Tang et al., 2019). When tested as a synthetic compound in our experiments, dimethyl trisulfide was the most toxic molecule amongst those specifically emitted on LBA (Table 2). It should be noted that *H. abietinum* was particularly sensitive to dimethyl trisulfide (MIC = 1 μL) as compared with the results reported on *S. sclerotiorum* (MIC = 24.04 μL) by Giorgio et al. (2015). Dimethyl disulfide has been frequently found in the volatilome of *Pseudomonas* spp. strains capable to antagonize diverse soil-borne fungal and oomycete pathogens (Giorgio et al., 2015; Ossowicki et al., 2017; Guevara-Avenda[~no et al., 2019; Báez-Vallejo et al., 2020). In the *Pseudomonas* genus, the production of volatiles such as dimethyl sulfide, dimethyl trisulfide, *S*-methyl thioacetate, methyl thiocyanate, 1-undecan, and HCN is a conserved trait and depends on the GacS/GacA two-component regulatory system (Cheng et al., 2016; Ossowicki et al., 2017). It is worthy of noting that dimethyl disulfide has been used as a pre-plant soil fumigant against nematodes, weeds, and soil-borne plant pathogens (Wang et al., 2009; Leocata et al., 2014; Mao et al., 2019; Yan et al., 2019). It acts by inducing mitochondria dysfunction, activation of ATP-sensitive potassium channels, and inhibition of cytochrome oxidase (Auger et al., 1989; Van Wambeke et al., 2009). Intriguingly, in our RNA-Seq experiment, several genes related to these processes had significantly altered expression: 51 genes related to mitochondria, two related to the potassium channel, and 23 cytochrome oxidases (i.e., cytochrome P450 and cytochrome-c). It has been also demonstrated that dimethyl disulfide may exert either phytotoxic activity (Kai et al., 2010; Tyagi et al., 2018) or promote plant growth (Groenhagen et al., 2013; Meldau et al., 2013) and, in some cases, induce systemic resistance (Tyagi et al., 2020). Phytotoxicity of sulfur-containing VOCs has not been infrequent though it is not a general rule (Chinchilla et al., 2019). Like for ammonia, cell membranes are permeable to VOCs (Pichersky et al., 2006) and frequently found injured upon exposure to dimethyl disulfide, dimethyl trisulfide, and other VOCs (Giorgio et al., 2015; Tang et al., 2019; Tyagi et al., 2020). Deterioration of subcellular structures such as Golgi bodies and mitochondria occurs subsequently, thus promoting vacuole formation in fungi (Tang et al., 2019). In *C. gloeosporioides*, membrane alteration induced by dimethyl trisulfide co-occurred with the down-regulation of genes related to the biosynthesis of β-1,3-D-glucan and chitin, which are components of the cell wall (Tang et al., 2019). In our data, the expression level of genes devoted to the same function in *H. abietinum* was not altered. Similarly, in bacteria, quaternary ammonium compounds disinfectants have been proved to disrupt the cell wall and leaking of the cytoplasm (Bragg et al., 2014), indicating that these cell injuries might be non-specific. Based on our SEM and TEM observations, *H. abietinum* plasma membrane was broken as early as 15 min of exposure to bacterial VOCs with subsequent repercussions on the inner cell content (Figures 6, 7). In the early stage of VOC exposure, the cell wall remained intact but collapsed later on and gave a shrunk aspect to the hyphae. This aspect has been also observed during the interaction of dimethyl trisulfide with *C. gloeosporioides* (Tang et al., 2019).

**FIGURE 9:**
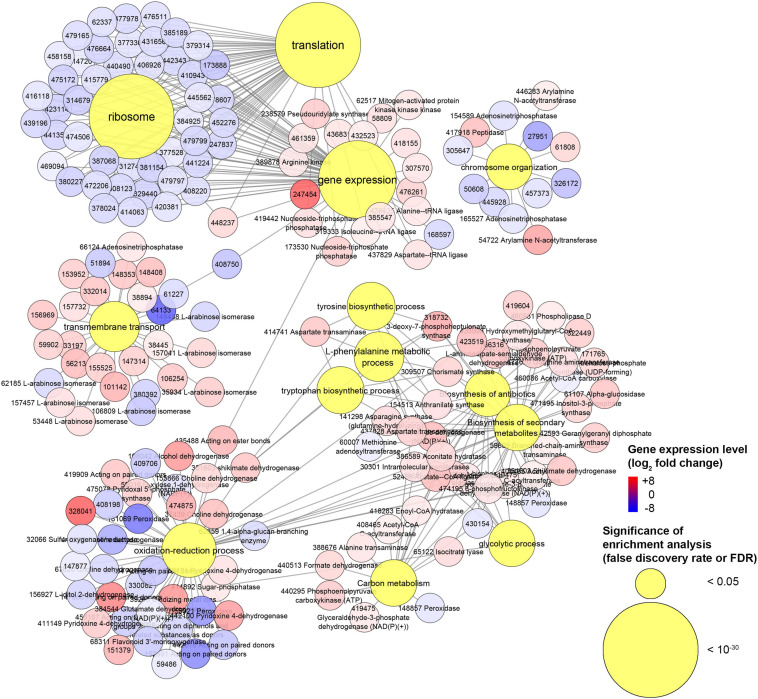
*Experiment L.* Network of genes associated with gene ontologies (GOs) which were significant (false discovery rate or FDR < 0.05) in the enrichment analysis. This is a subnetwork manually extracted from the network depicted in Supplementary Figure 13 to show GOs with the most contrasting gene regulation.

Our transcriptomics analysis on *H. abietinum* revealed clear detrimental effects on the ribosomes and translation (Figures 8, 9), though it could not be sure if they were VOC-specific mechanisms or just an overall consequence of the compromised growth. Nevertheless, similar perturbations have occurred in *P. infestans* exposed to sulfur-containing VOCs (Chinchilla et al., 2019). Also, several similarities have been found in another work that investigated the dimethyl disulfide toxicity to *C. gloeosporioides*: GOs related to ribosomes, cellular membranes, organelles, transporter activity, secondary metabolites, carbohydrate metabolism, tryptophan, and tyrosine biosynthesis were all enriched (Tang et al., 2019). Similarly, we also found induction of the biosynthesis of cysteine, tyrosine, tryptophan, and L-phenylalanine. This might be seen as incongruent with the inhibition of the translation mentioned above. However, these biosynthetic processes are closely linked with the shikimate pathway (Maeda and Dudareva, 2012), which is upstream of the biosynthesis of numerous aromatic secondary metabolites such as alkaloids, flavonoids, lignins, and antibiotics (Tohge et al., 2013). Many of these compounds play important roles in the defense against biotic and abiotic stresses (Maeda and Dudareva, 2012). Although in our study the secondary metabolites pathway was induced (Figure 8), *terpene cyclase* and *dimethylallyltransferase synthase-type prenyltransferase* genes coding for the enzymes required for fomannosin and fomannoxin biosynthesis (the main toxins of *H. annosum* s. l.) (Olson et al., 2012; Antipova et al., 2020) were not differentially regulated. The high demand for carbon from the shikimate pathway (Ni et al., 1996), especially under stress conditions (Corea et al., 2012), could explain why we found up-regulation of the carbon metabolism (Figure 8). Most of these pathways are interconnected: for example, genes like *shikimate dehydrogenase*, *chorismate synthase*, and *3-deoxy-7-phosphoheptulonate synthase* work both in the shikimate pathways and oxidation-reduction processes.

## Conclusion

Volatile compounds emitted by numerous bacteria inhibited completely the growth of almost all the Basidiomycete species tested, including *H. abietinum*, which was used in this research as a model species. This finding paves new ways for the management of Basidiomycete-induced diseases in both agricultural and forestry systems.

Digested proteins like peptone or tryptone were nutrients required for the production of such inhibitory volatiles. In the volatilome of *P. protegens* CHA0, ammonia played an important role in the *H. abietinum* growth inhibition but was not the only antifungal compound. Three aspects led to think that ammonia was among the most effective substances: (a) it was highly toxic (EC_50_ = 1.18 M; MIC = 2.14 M) when tested as pure compound (ammonium hydroxide); (b) its vapors induced a pH increase of the agar media, a phenomenon also observed upon the exposure to the CHA0’s volatiles; and (c) the high toxicity of ammonia could explain why urea, which is converted by ureases into ammonia and carbamic acid, has been widely used on large scale against *Heterobasidion* spp. infections in forest trees.

At least two aspects, however, supported the participation of additional VOCs: (a) other compounds emitted by CHA0 grown in media containing digested proteins (e.g., Luria-Bertani agar) were even more toxic than ammonia against *H. abietinum* (e.g., dimethyl trisulfide had EC_50_ = 0.02 M and MIC = 0.2 M, and 2-ethylhexanol had EC_50_ = 0.33 M and MIC = 0.77 M); and (b) some transcriptional changes of *H. abietinum* were similar to those observed in *P. infestans* exposed to sulfur-containing VOCs (Chinchilla et al., 2019) or in *C. gloeosporioides* exposed to dimethyl disulfide (Tang et al., 2019).

Bacterial volatiles caused severe cellular and sub-cellular alterations of *H. abietinum* hyphae as early as 15 min of exposure to VOCs, as evidenced by transmission and scanning electron microscopy observations.

Analysis of the transcriptome reprogramming of *H. abietinum* induced by CHA0’s VOCs pointed out that detrimental effects occurred on ribosomes and protein synthesis while the cells probably tried to react by activating defense mechanisms and thus consumed a lot of energy diverted from the growth and development (fitness cost).

Future research could unveil the reason why the sensitivity to bacterial volatiles was widespread among Basidiomycetes while resistance was observed only in some Ascomycete strains. It could be interesting to investigate whether a difference in permeability of the plasma membrane or cell wall is associated with the sensitivity/resistance. Finally, the specific molecular sites of action of the antifungal volatiles merit to be precisely identified.

## Data Availability Statement

The datasets presented in this study can be found in online repositories. The names of the repository/repositories and accession number(s) can be found below: https://www.ncbi.nlm.nih.gov/bioproject/PRJNA716634.

## Author Contributions

GB and MP conceived the research. MP performed the *in vitro* experiments. GB analyzed the transcriptomics data, carried out the statistical analysis, and prepared tables and graphs. FM provided and handled the Basidiomycete isolates. AS made observations with the transmission electron microscope. AA, FL’H, and LW performed the analysis of volatile organic compounds. GB wrote the manuscript. All the authors revised the manuscript. All authors contributed to the article and approved the submitted version.

## Conflict of Interest

The authors declare that the research was conducted in the absence of any commercial or financial relationships that could be construed as a potential conflict of interest.

## Footnotes

1 http://www.bioinformatics.babraham.ac.uk/projects/fastqc/

2 https://genome.jgi.doe.gov/portal/Hetan2/Hetan2.download.html

3 http://www.Rproject.org

4 http://www.rstudio.com

## Supplementary Material

Supplementary Figure 1 Phylogenetic analysis of 63 isolates belonging to the *Heterobasidion* genus, including the strain 10 used in this study, computed by the maximum likelihood method and based on four concatenated genes. The genes used were: *glutathione-S-transferase 1* (GST1), *elongation factor 1-*α (EFA), *glyceraldehyde 3-phosphate dehydrogenase* (G3P), and a transcription factor (TF). The percentage of trees (out of 1000 bootstraps) in which the associated taxa clustered together is shown next to the branches. There were a total of 1171 sites in the final dataset. The tree is drawn to scale, with branch lengths measured in the number of substitutions per site. The accession numbers of the isolates are given in Supplementary Table 1.

Supplementary Figure 2 *Experiment B.* Effect of volatile organic compounds (VOCs) emitted by several bacteria grown on Luria-Bertani agar (LBA) against *Heterobasidion abietinum* strain 10. The photos show colonies of *Heterobasidion abietinum* strain 10 after 7 days of exposure to bacterial VOCs in the overlapping plate assay. Halos around the colonies were due to diffusible substances secreted by the fungus and did not contain hyphae. Each plate is relative to one bacterial strain and representative of three replicates. In the graph, error bars are the standard error of the mean of three replicates (plates).

Supplementary Figure 3 *Supplementary Experiment 1*. In the overlapping plate assay, few colonies of *Pseudomonas protegens* CHA0 were enough to inhibit the growth of *Heterobasidion abietinum* strain 10. Halos around the fungal colonies were due to diffusible substances secreted by the fungus and did not contain hyphae. In the graph, error bars are the standard error of the mean of three replicates (plates). Bars with different letters are significantly different according to the LSD test (*P* < 0.05).

Supplementary Figure 4 *Supplementary Experiment 2.* Two methods for testing the production of hydrogen cyanide (HCN) by *Pseudomonas protegens* strains CHA0 and CHA77. The color of the filter paper in the plate lid, which was above the plate with the bacteria for 7 days, indicated the production of HCN by CHA0, but not CHA77, which is its deficient mutant. Method 1 was based on copper(II) ethylacetoacetate and 4,4′-methylenebis-(*N*,*N*-dimethylaniline) (Castric and Castric, 1983; Voisard et al., 1989). Method 2 was based on picric acid and sodium carbonate (Bakker and Schippers, 1987). The bacteria were grown on Luria-Bertani agar.

Supplementary Figure 5 Effect of volatile organic compounds (VOCs) emitted by several bacteria grown on Luria-Bertani agar (LBA) or potato dextrose agar (PDA) against *Heterobasidion abietinum* strain 10. The photos show colonies of *Heterobasidion abietinum* strain 10 after 7 days of exposure to bacterial VOCs in the overlapping plate assay. Halos around the colonies were due to diffusible substances secreted by the fungus and did not contain hyphae. In the graphs, error bars are the standard error of the mean of three replicates (plates). Bars with the asterisk are significantly different from the control according to the LSD test (*P* < 0.05).

Supplementary Figure 6 *Supplementary Experiment 3*. Evaluation of ammonia production by *Pseudomonas protegens* strains CHA0, CHA77, and other bacteria grown in diverse media. Two hundred fifty mL of Nessler’s reagent were added to 48 h-liquid cultures (tube on the left side of each picture) and the resulting color was compared with the untreated control (tube on the right side); the yellow color, and possibly the formation of precipitates, is indicative of ammonia production.

Supplementary Figure 7 *Experiment G.* Volatilome analysis of *Pseudomonas protegens* CHA0 grown on Luria-Bertani agar (LBA) or potato dextrose agar (PDA) for 48 h with uninoculated media as blank controls. **(A)** Principal component analysis (PCA). **(B)** Heatmap of mass features. **(C)** Significant mass features (mz/rt) represented with red dots and identified by one-way ANOVA followed by *post hoc* test (*P* < 0.05).

Supplementary Figure 8 *Experiment L.* Principal component analysis (PCA; **A**) and the mean-difference plot **(B)** for the RNA-Seq experiment where *Heterobasidion abietinum* strain 10 was exposed to the volatile organic compounds (VOCs) emitted by *Pseudomonas protegens* CHA0 grown on Luria-Bertani agar (LBA) versus potato dextrose agar (PDA).

Supplementary Figure 9 *Experiment L.* Enriched (false discovery rate or FDR < 0.05) gene ontology terms of the down-regulated genes identified in the RNA-Seq experiment. For better readability, only representative branch nodes are showed.

Supplementary Figure 10 *Experiment L.* Enriched (false discovery rate or FDR < 0.05) gene ontology terms of the up-regulated genes identified in the RNA-Seq experiment. For better readability, only representative branch nodes are showed.

Supplementary Figure 11 *Experiment L.* KEGG pathway hir03010 (ribosome) with genes detected in the RNA-Seq experiment. Eight down-regulated genes (highlighted in blue) and 30 genes with unaltered expression (highlighted in gray) were associated with this pathway.

Supplementary Figure 12 *Experiment L.* KEGG pathway hir00400 (phenylalanine, tyrosine, and tryptophan biosynthesis) with genes detected in the RNA-Seq. Nine up-regulated genes (highlighted in red) and 10 genes with unaltered expression (highlighted in gray) were associated with this pathway.

Supplementary Figure 13 *Experiment L.* Networks constructed with genes associated with gene ontologies (GOs) of biological process, molecular function, and cellular component, as well as the KEGG pathways which were significant (false discovery rate or FDR < 0.05) in the enrichment analyses.

Supplementary Table 1 Accession numbers of the genes used for the phylogenetic analysis showed in Supplementary Figure 1.

Supplementary Table 2 *Experiment L.* Expression levels of genes identified by RNA-Seq in *H. abietinum* strain 10 exposed for 12 h to volatile organic compounds (VOCs) emitted by *P. protegens* CHA0 grown on Luria-Bertani agar (LBA) or, as control, potato dextrose agar (PDA). Gene annotations with proteins, enzyme codes, and gene ontologies are also reported. Genes with a false discovery rate (FDR) lower than 0.05 were considered (significantly) differentially expressed. Among these genes, those with an expression level (expressed as log_2_ fold change or log_2_FC) higher or lower than zero were considered up- or down-regulated, respectively.

Supplementary Table 3 *Experiment L.* Over-representation analysis (false discovery rate or FDR < 0.05) of the gene ontology (GO) terms associated with the differentially expressed genes (DEGs) identified in the RNA-Seq experiment.

Supplementary Experiment 1 Magnitude of CHA0’s effect against *H. abietinum* growth.

Supplementary Experiment 2 Testing the production of hydrogen cyanide (HCN) by *Pseudomonas protegens* strains CHA0 and CHA77.

Supplementary Experiment 3 Evaluation of ammonia production by bacteria.
