# Supplementary material for: Basidiomycetes Are Particularly Sensitive to Bacterial Volatile Compounds: Mechanistic Insight Into the Case Study of Pseudomonas protegens Volatilome Against Heterobasidion abietinum
Source: Front Microbiol. 2021 May 31;12:684664. doi: 10.3389/fmicb.2021.684664 (PMC8248679; doi:10.3389/fmicb.2021.684664)
Supplement: Supplementary Figure 1 — Phylogenetic analysis of 63 isolates belonging to the Heterobasidion genus, including the strain 10 used in this study, computed by the maximum likelihood method and based on four concatenated genes. The genes used were: glutathione-S-transferase 1 (GST1), elongation factor 1-α (EFA), glyceraldehyde 3-phosphate dehydrogenase (G3P), and a transcription factor (TF). The percentage of trees (out of 1000 bootstraps) in which the associated taxa clustered together is shown next to the branches. There were a total of 1171 sites in the final dataset. The tree is drawn to scale, with branch lengths measured in the number of substitutions per site. The accession numbers of the isolates are given in Supplementary Table 1. [file Presentation_1.zip › Supplementary material/Supplementary Experiment 1.pdf]

Supplementary Experiment 1. Magnitude of CHA0's effect against *H. abietinum* growth.

In OP assays, a different number of bacterial colonies were tested to understand how many colonies could produce a VOC amount sufficient for the inhibition. Bacterial colonies were picked from 48 h-LBA plates using a needle loop and point-inoculated onto fresh LBA for the OP assay. Although they did not originate from single cells, they were considered single colonies because confined at the point of inoculation. Plates with 1, 2, 3, 4, 5, and 9 colonies were prepared by this procedure. Also, other plates were prepared by spreading 100 µL 24 h-LB cultures with 10^3 and 10^6 cells mL^{-1} onto the agar surface using a glass rod. Therefore, eight plates with a different number of colonies were prepared and three replicates were used. Plates without bacteria served as control.

Results are shown in **Supplementary Figure 3**.
