# Supplementary material for: Basidiomycetes Are Particularly Sensitive to Bacterial Volatile Compounds: Mechanistic Insight Into the Case Study of Pseudomonas protegens Volatilome Against Heterobasidion abietinum
Source: Front Microbiol. 2021 May 31;12:684664. doi: 10.3389/fmicb.2021.684664 (PMC8248679; doi:10.3389/fmicb.2021.684664)
Supplement: Supplementary Figure 1 — Phylogenetic analysis of 63 isolates belonging to the Heterobasidion genus, including the strain 10 used in this study, computed by the maximum likelihood method and based on four concatenated genes. The genes used were: glutathione-S-transferase 1 (GST1), elongation factor 1-α (EFA), glyceraldehyde 3-phosphate dehydrogenase (G3P), and a transcription factor (TF). The percentage of trees (out of 1000 bootstraps) in which the associated taxa clustered together is shown next to the branches. There were a total of 1171 sites in the final dataset. The tree is drawn to scale, with branch lengths measured in the number of substitutions per site. The accession numbers of the isolates are given in Supplementary Table 1. [file Presentation_1.zip › Supplementary material/Supplementary Experiment 3.pdf]

Supplementary Experiment 3. Evaluation of ammonia production by bacteria.

Ammonia production by bacteria was tested according to Cappuccino and Welsh (2019). The strains CHA0 and CHA77 were included in this assay, along with the laboratory strain *Escherichia coli* TG1 and three plant pathogens such as *Pectobacterium carotovorum* subsp. *carotovorum* NCPPB 312 (type strain of the species), and *Pseudomonas syringae* pv. *tomato*; *H. abietinum* strain 10 was also included as a negative control. Microorganisms were grown in test tubes containing 10 mL of different liquid media, including peptone water (PW; 10 g L^{-1} Bacto™ proteose peptone, 5 g L^{-1} NaCl, pH 7.2), Luria-Bertani broth (LB), potato dextrose broth (PD), peptone broth (P; 10 g L^{-1} Bacto™ proteose peptone), and King's B broth (KB). After 48 h incubation at 27 °C and 250 rpm, 250 mL Nessler's reagent (CAS no. 7783-33-7, Carlo Erba Reagents) were added to a 4 mL culture aliquot transferred to another test tube. The development of yellow to brownish color of the culture indicated the ammonia production.

Both CHA0 and CHA77, along with three other bacteria tested in this experiment, were proved to produce ammonia (**Supplementary Figure 6**).

References

Cappuccino, J. G., and Welsh, C. (2019). Microbiology: a laboratory manual, eds. J.G. Cappuccino, and C. Welsh Twelfth ed (New York, USA: Pearson), 541 pp.
