# Supplementary material for: Basidiomycetes Are Particularly Sensitive to Bacterial Volatile Compounds: Mechanistic Insight Into the Case Study of Pseudomonas protegens Volatilome Against Heterobasidion abietinum
Source: Front Microbiol. 2021 May 31;12:684664. doi: 10.3389/fmicb.2021.684664 (PMC8248679; doi:10.3389/fmicb.2021.684664)
Supplement: Supplementary Figure 1 — Phylogenetic analysis of 63 isolates belonging to the Heterobasidion genus, including the strain 10 used in this study, computed by the maximum likelihood method and based on four concatenated genes. The genes used were: glutathione-S-transferase 1 (GST1), elongation factor 1-α (EFA), glyceraldehyde 3-phosphate dehydrogenase (G3P), and a transcription factor (TF). The percentage of trees (out of 1000 bootstraps) in which the associated taxa clustered together is shown next to the branches. There were a total of 1171 sites in the final dataset. The tree is drawn to scale, with branch lengths measured in the number of substitutions per site. The accession numbers of the isolates are given in Supplementary Table 1. [file Presentation_1.zip › Supplementary material/Supplementary Table 1.pdf]

Supplementary Table 1. Accession numbers of the genes used for the phylogenetic analysis showed in Supplementary Figure 1.

Species and isolate	Genes			
	**GST1**	**EFA**	**G3P**	**TF**
H. abietinum isolate 10	MZ322410	MZ322411	MZ322412	MZ322413
H. abietinum isolate 87064/2	FJ627335.1	FJ627370.1	FJ627441.1	FJ627733.1
H. abietinum isolate 92137/4	FJ627337.1	FJ627378.1	FJ627451.1	FJ627769.1
H. abietinum isolate 92144/1	FJ627346.1	FJ627379.1	FJ627452.1	FJ627770.1
H. abietinum isolate 92179/3	FJ627338.1	FJ627382.1	FJ627456.1	FJ627774.1
H. abietinum isolate FA8	FJ627340.1	FJ627399.1	FJ627479.1	FJ627735.1
H. abietinum isolate FB18	FJ627343.1	FJ627410.1	FJ627490.1	FJ627738.1
H. abietinum isolate Faf4-2	FJ627345.1	FJ627400.1	FJ627480.1	FJ627739.1
H. abietinum isolate Faf4-6	FJ627339.1	FJ627401.1	FJ627481.1	FJ627734.1
H. abietinum isolate Faf5-3	FJ627341.1	FJ627402.1	FJ627482.1	FJ627736.1
H. abietinum isolate Faf6-3	FJ627347.1	FJ627403.1	FJ627483.1	FJ627740.1
H. abietinum isolate Faf7-2	FJ627342.1	FJ627404.1	FJ627484.1	FJ627737.1
H. annosum isolate 90211/2	FJ627302.1	FJ627374.1	FJ627446.1	FJ627764.1
H. annosum isolate 90231/2	FJ627311.1	FJ627375.1	FJ627447.1	FJ627765.1
H. annosum isolate 91202/2	FJ627303.1	FJ627377.1	FJ627449.1	FJ627767.1
H. annosum isolate 92153/1	FJ627304.1	FJ627380.1	FJ627453.1	FJ627771.1
H. annosum isolate 92181/5	FJ627357.1	FJ627383.1	FJ627457.1	FJ627716.1
H. annosum isolate 92182/1	FJ627313.1	FJ627384.1	FJ627458.1	FJ627717.1
H. annosum isolate 93014/1	FJ627305.1	FJ627385.1	FJ627459.1	FJ627775.1
H. annosum isolate AR18	FJ627306.1	FJ627393.1	FJ627471.1	FJ627709.1
H. annosum isolate AR19	FJ627318.1	FJ627394.1	FJ627472.1	FJ627722.1
H. annosum isolate Bc-4b-2	FJ627291.1	FJ627397.1	FJ627477.1	FJ627699.1
H. annosum isolate Bc-5b-15	FJ627292.1	FJ627398.1	FJ627478.1	FJ627700.1
H. annosum isolate FW2-2	FJ627307.1	FJ627411.1	FJ627491.1	FJ627710.1
H. annosum isolate Faf10-7	FJ627293.1	FJ627405.1	FJ627485.1	FJ627701.1
H. annosum isolate MJT272	FJ627308.1	FJ627415.1	FJ627496.1	FJ627711.1
H. annosum isolate MON108	FJ627294.1	FJ627416.1	FJ627497.1	FJ627702.1
H. annosum isolate MON109	FJ627295.1	FJ627417.1	FJ627498.1	FJ627703.1
H. annosum isolate MON111	FJ627287.1	FJ627418.1	FJ627499.1	FJ627695.1
H. annosum isolate ORE102	FJ627296.1	FJ627419.1	FJ627503.1	FJ627704.1
H. annosum isolate ORE103	FJ627297.1	FJ627420.1	FJ627504.1	FJ627705.1
H. annosum isolate ORE104	FJ627282.1	FJ627421.1	FJ627505.1	FJ627690.1
H. annosum isolate Sa16-4	FJ627309.1	FJ627422.1	FJ627507.1	FJ627712.1
H. annosum isolate TC111-3	FJ627288.1	FJ627426.1	FJ627511.1	FJ627696.1
H. annosum isolate TC111-4	FJ627289.1	FJ627427.1	FJ627512.1	FJ627697.1
H. annosum isolate TC122-11	FJ627299.1	FJ627428.1	FJ627513.1	FJ627707.1
H. annosum isolate TC122-12	FJ627298.1	FJ627429.1	FJ627514.1	FJ627706.1
H. annosum isolate TC32-1	FJ627283.1	FJ627423.1	FJ627508.1	FJ627691.1
H. annosum isolate TC32-7	FJ627284.1	FJ627424.1	FJ627509.1	FJ627692.1
H. annosum isolate TC39-7	FJ627285.1	FJ627425.1	FJ627510.1	FJ627693.1
H. annosum isolate V13:53-c2	FJ627312.1	FJ627432.1	FJ627517.1	FJ627714.1
H. annosum isolate V3:47-C4	FJ627315.1	FJ627430.1	FJ627515.1	FJ627719.1
H. annosum isolate V5:91-C4	FJ627316.1	FJ627431.1	FJ627516.1	FJ627720.1
H. annosum isolate VMTB3	FJ627286.1	FJ627364.1	FJ627435.1	FJ627694.1
H. annosum isolate W15	FJ627310.1	FJ627434.1	FJ627519.1	FJ627713.1
H. parviporum isolate 87-124-2	FJ627330.1	FJ627365.1	FJ627436.1	FJ627753.1
H. parviporum isolate 87-176/2	FJ627331.1	FJ627367.1	FJ627438.1	FJ627755.1
H. parviporum isolate 87075/2	FJ627349.1	FJ627371.1	FJ627444.1	FJ627762.1
H. parviporum isolate 90166/2	FJ627320.1	FJ627373.1	FJ627445.1	FJ627763.1
H. parviporum isolate 93026/1	FJ627322.1	FJ627386.1	FJ627460.1	FJ627776.1

**Species and isolate**	**Genes**			
	**GST1**	**EFA**	**G3P**	**TF**
H. parviporum isolate 93028/1	FJ627319.1	FJ627387.1	FJ627461.1	FJ627724.1
H. parviporum isolate 93134/1	FJ627323.1	FJ627388.1	FJ627463.1	FJ627726.1
H. parviporum isolate 95151	FJ627326.1	FJ627389.1	FJ627468.1	FJ627728.1
H. parviporum isolate 95156	FJ627325.1	FJ627390.1	FJ627469.1	FJ627727.1
H. parviporum isolate 95191	FJ627329.1	FJ627392.1	FJ627470.1	FJ627730.1
H. parviporum isolate B1295	FJ627359.1	FJ627395.1	FJ627475.1	FJ627749.1
H. parviporum isolate B1314	FJ627360.1	FJ627396.1	FJ627476.1	FJ627750.1
H. parviporum isolate Fas1	FJ627327.1	FJ627406.1	FJ627486.1	FJ627729.1
H. parviporum isolate Fas10	FJ627351.1	FJ627407.1	FJ627487.1	FJ627742.1
H. parviporum isolate Fas11	FJ627350.1	FJ627408.1	FJ627488.1	FJ627741.1
H. parviporum isolate Fas13	FJ627354.1	FJ627409.1	FJ627489.1	FJ627746.1
H. parviporum isolate HR20	FJ627352.1	FJ627413.1	FJ627493.1	FJ627744.1
H. parviporum isolate HR32	FJ627353.1	FJ627414.1	FJ627494.1	FJ627745.1

GST1: *glutathione-S-transferase 1*; EFA: *elongation factor 1-α*; G3P: *glyceraldehyde 3-phosphate dehydrogenase*; TF: transcription factor.
