# Supplementary figures and images for: Basidiomycetes Are Particularly Sensitive to Bacterial Volatile Compounds: Mechanistic Insight Into the Case Study of Pseudomonas protegens Volatilome Against Heterobasidion abietinum
Source: Front Microbiol. 2021 May 31;12:684664. doi: 10.3389/fmicb.2021.684664 (PMC8248679; doi:10.3389/fmicb.2021.684664)

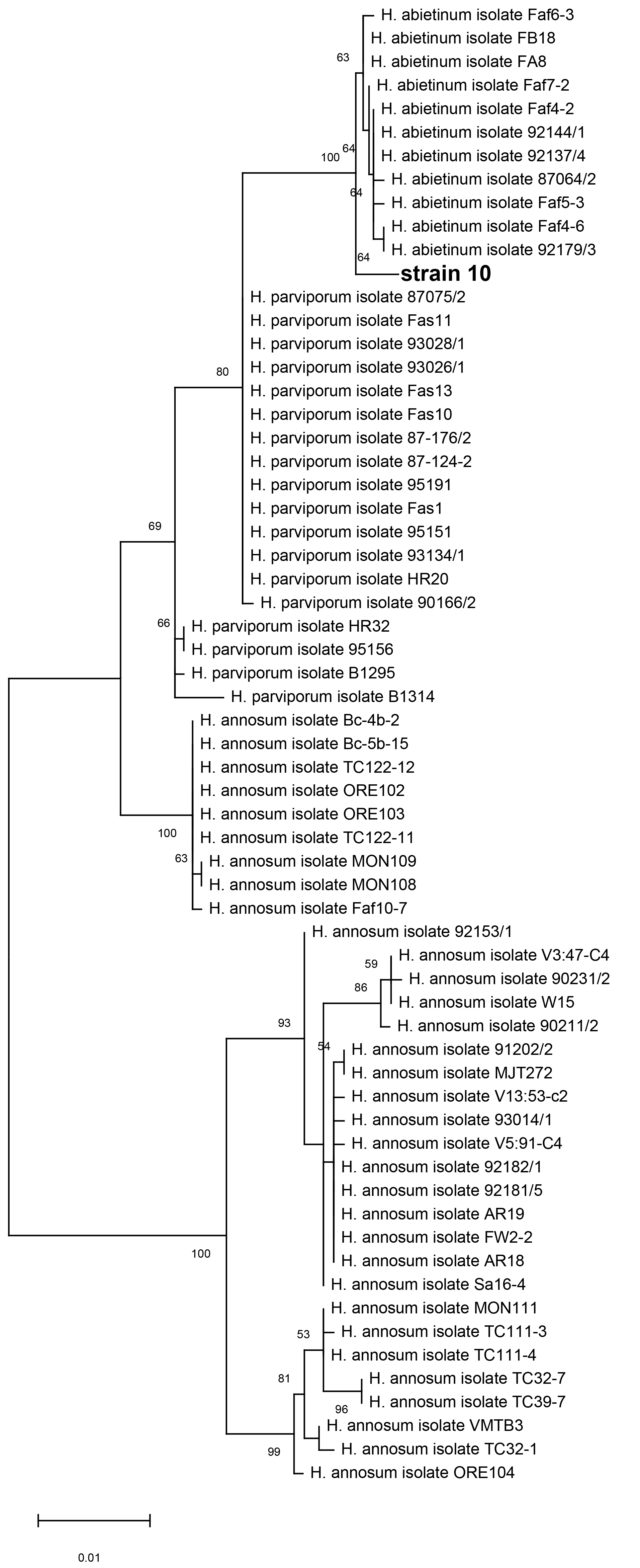

Supplement: Supplementary Figure 1 — Phylogenetic analysis of 63 isolates belonging to the Heterobasidion genus, including the strain 10 used in this study, computed by the maximum likelihood method and based on four concatenated genes. The genes used were: glutathione-S-transferase 1 (GST1), elongation factor 1-α (EFA), glyceraldehyde 3-phosphate dehydrogenase (G3P), and a transcription factor (TF). The percentage of trees (out of 1000 bootstraps) in which the associated taxa clustered together is shown next to the branches. There were a total of 1171 sites in the final dataset. The tree is drawn to scale, with branch lengths measured in the number of substitutions per site. The accession numbers of the isolates are given in Supplementary Table 1. [file Presentation_1.zip › Supplementary material/Supplementary Figure 1.TIF]

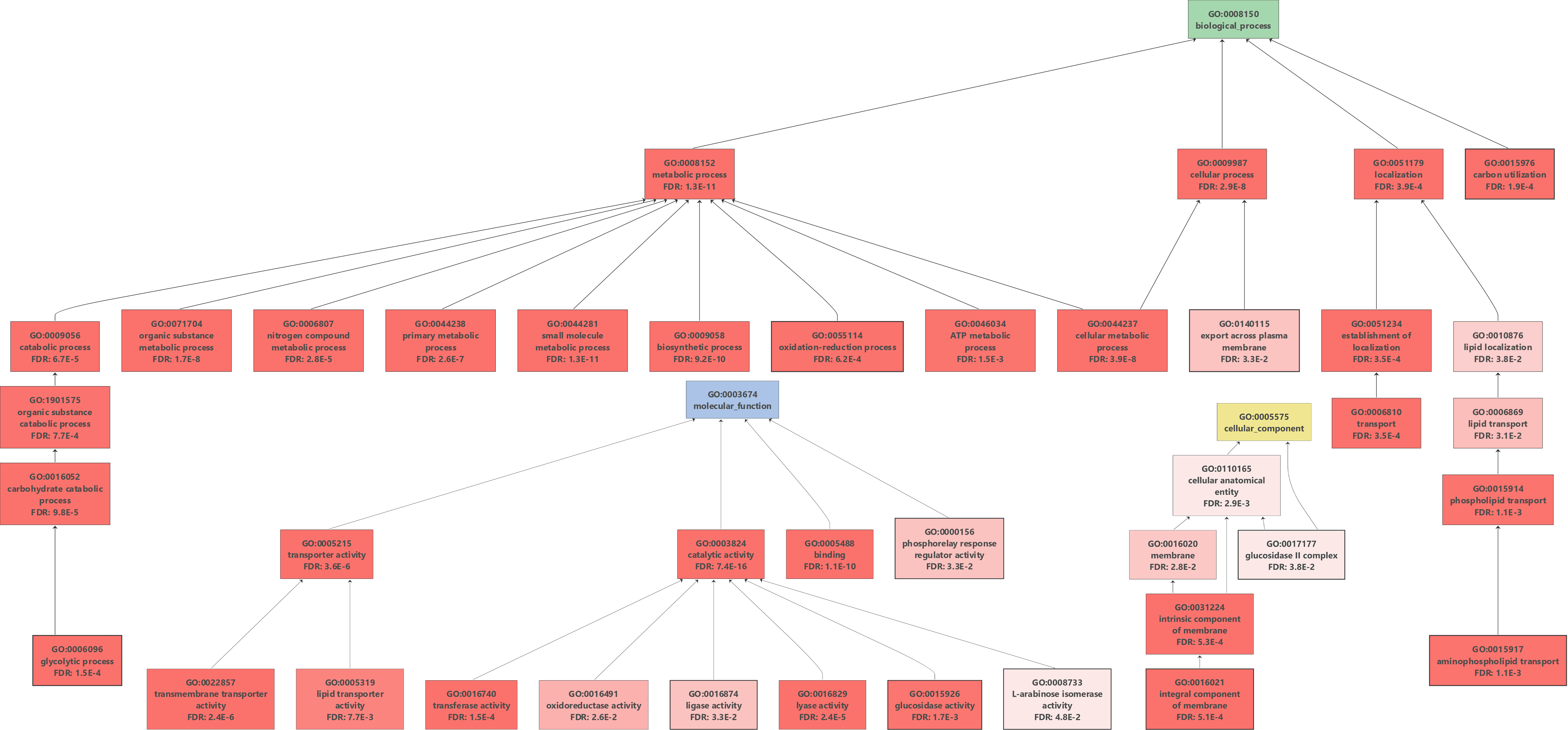

Supplement: Supplementary Figure 1 — Phylogenetic analysis of 63 isolates belonging to the Heterobasidion genus, including the strain 10 used in this study, computed by the maximum likelihood method and based on four concatenated genes. The genes used were: glutathione-S-transferase 1 (GST1), elongation factor 1-α (EFA), glyceraldehyde 3-phosphate dehydrogenase (G3P), and a transcription factor (TF). The percentage of trees (out of 1000 bootstraps) in which the associated taxa clustered together is shown next to the branches. There were a total of 1171 sites in the final dataset. The tree is drawn to scale, with branch lengths measured in the number of substitutions per site. The accession numbers of the isolates are given in Supplementary Table 1. [file Presentation_1.zip › Supplementary material/Supplementary Figure 10.TIF]

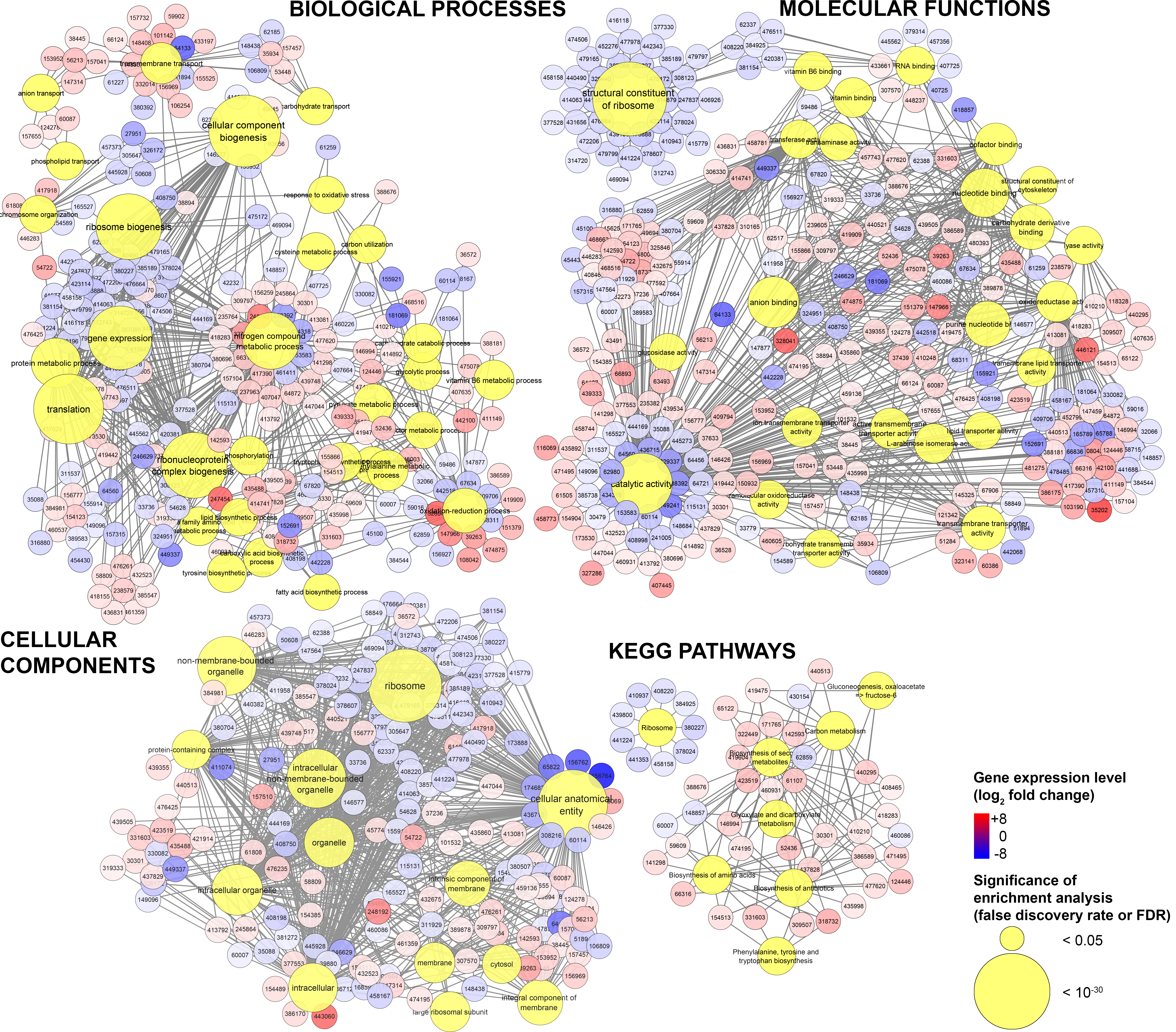

Supplement: Supplementary Figure 1 — Phylogenetic analysis of 63 isolates belonging to the Heterobasidion genus, including the strain 10 used in this study, computed by the maximum likelihood method and based on four concatenated genes. The genes used were: glutathione-S-transferase 1 (GST1), elongation factor 1-α (EFA), glyceraldehyde 3-phosphate dehydrogenase (G3P), and a transcription factor (TF). The percentage of trees (out of 1000 bootstraps) in which the associated taxa clustered together is shown next to the branches. There were a total of 1171 sites in the final dataset. The tree is drawn to scale, with branch lengths measured in the number of substitutions per site. The accession numbers of the isolates are given in Supplementary Table 1. [file Presentation_1.zip › Supplementary material/Supplementary Figure 13.TIF]

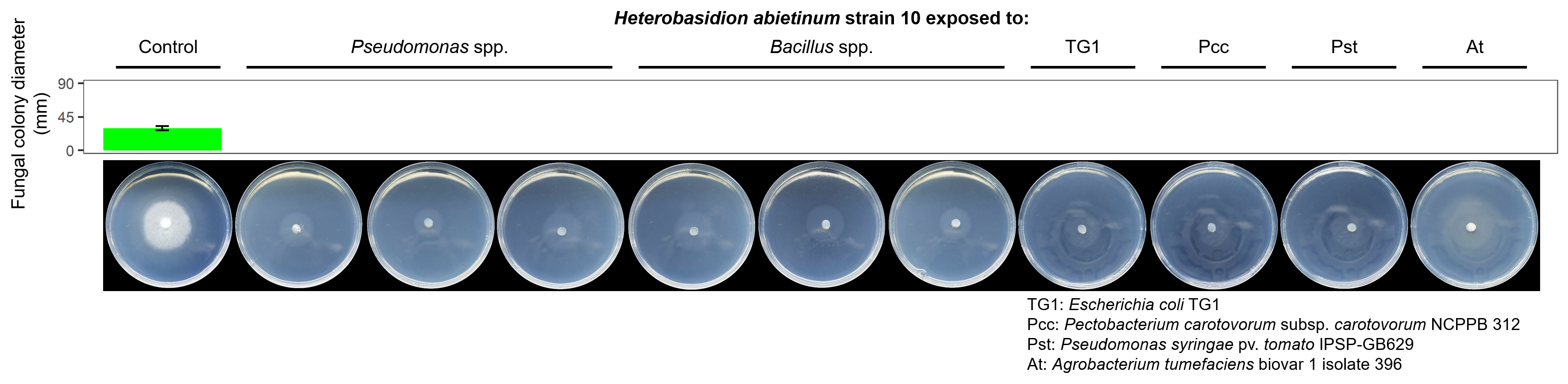

Supplement: Supplementary Figure 1 — Phylogenetic analysis of 63 isolates belonging to the Heterobasidion genus, including the strain 10 used in this study, computed by the maximum likelihood method and based on four concatenated genes. The genes used were: glutathione-S-transferase 1 (GST1), elongation factor 1-α (EFA), glyceraldehyde 3-phosphate dehydrogenase (G3P), and a transcription factor (TF). The percentage of trees (out of 1000 bootstraps) in which the associated taxa clustered together is shown next to the branches. There were a total of 1171 sites in the final dataset. The tree is drawn to scale, with branch lengths measured in the number of substitutions per site. The accession numbers of the isolates are given in Supplementary Table 1. [file Presentation_1.zip › Supplementary material/Supplementary Figure 2.TIF]

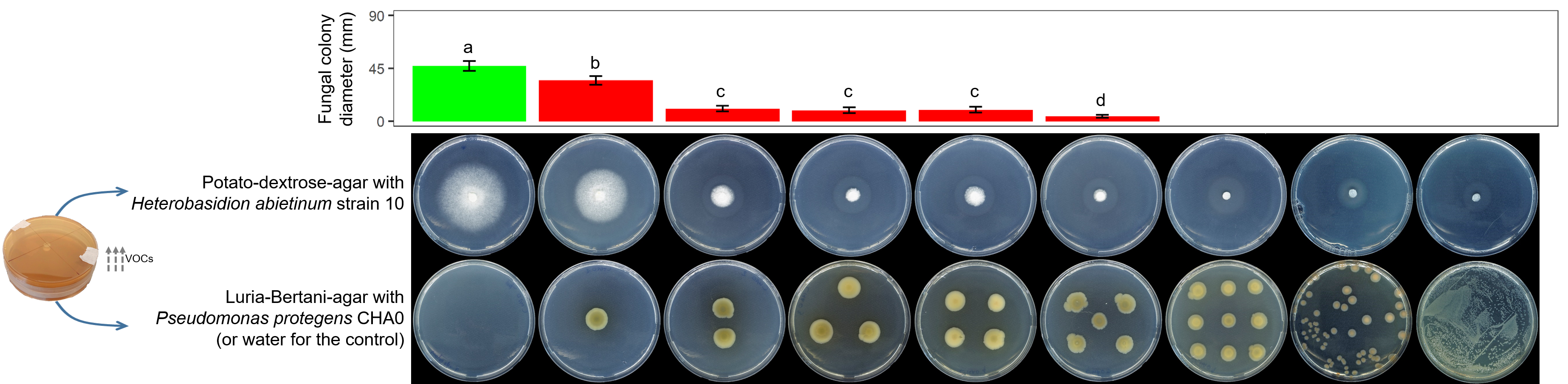

Supplement: Supplementary Figure 1 — Phylogenetic analysis of 63 isolates belonging to the Heterobasidion genus, including the strain 10 used in this study, computed by the maximum likelihood method and based on four concatenated genes. The genes used were: glutathione-S-transferase 1 (GST1), elongation factor 1-α (EFA), glyceraldehyde 3-phosphate dehydrogenase (G3P), and a transcription factor (TF). The percentage of trees (out of 1000 bootstraps) in which the associated taxa clustered together is shown next to the branches. There were a total of 1171 sites in the final dataset. The tree is drawn to scale, with branch lengths measured in the number of substitutions per site. The accession numbers of the isolates are given in Supplementary Table 1. [file Presentation_1.zip › Supplementary material/Supplementary Figure 3.TIF]

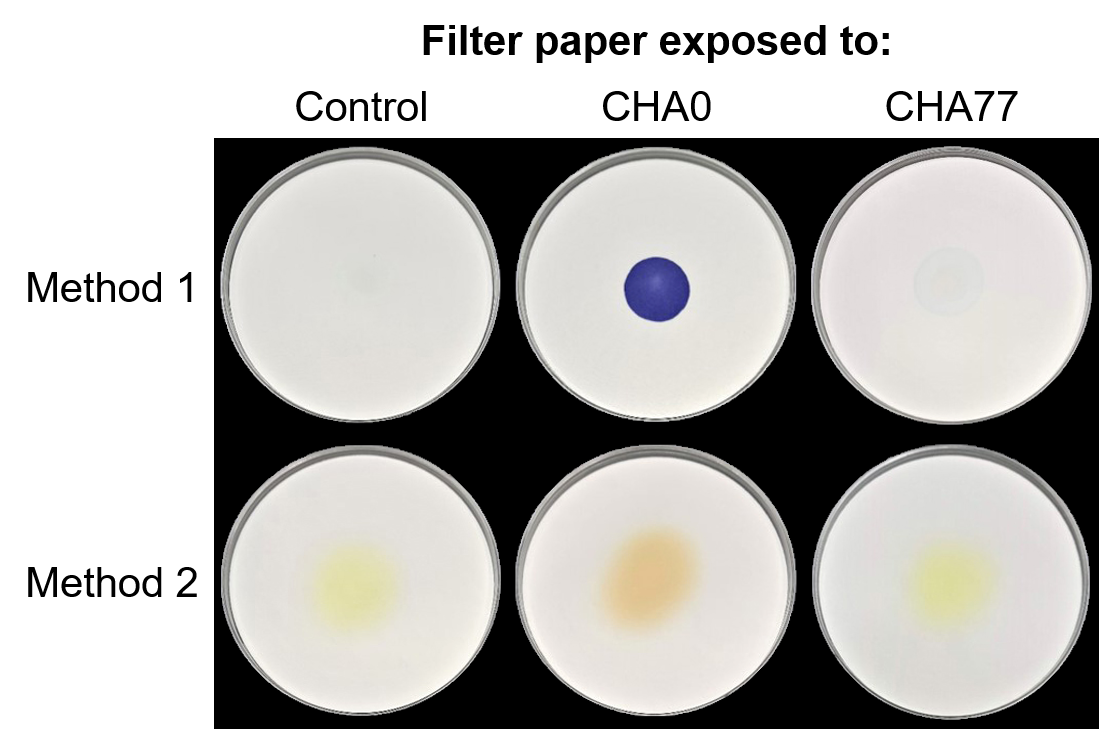

Supplement: Supplementary Figure 1 — Phylogenetic analysis of 63 isolates belonging to the Heterobasidion genus, including the strain 10 used in this study, computed by the maximum likelihood method and based on four concatenated genes. The genes used were: glutathione-S-transferase 1 (GST1), elongation factor 1-α (EFA), glyceraldehyde 3-phosphate dehydrogenase (G3P), and a transcription factor (TF). The percentage of trees (out of 1000 bootstraps) in which the associated taxa clustered together is shown next to the branches. There were a total of 1171 sites in the final dataset. The tree is drawn to scale, with branch lengths measured in the number of substitutions per site. The accession numbers of the isolates are given in Supplementary Table 1. [file Presentation_1.zip › Supplementary material/Supplementary Figure 4.TIF]

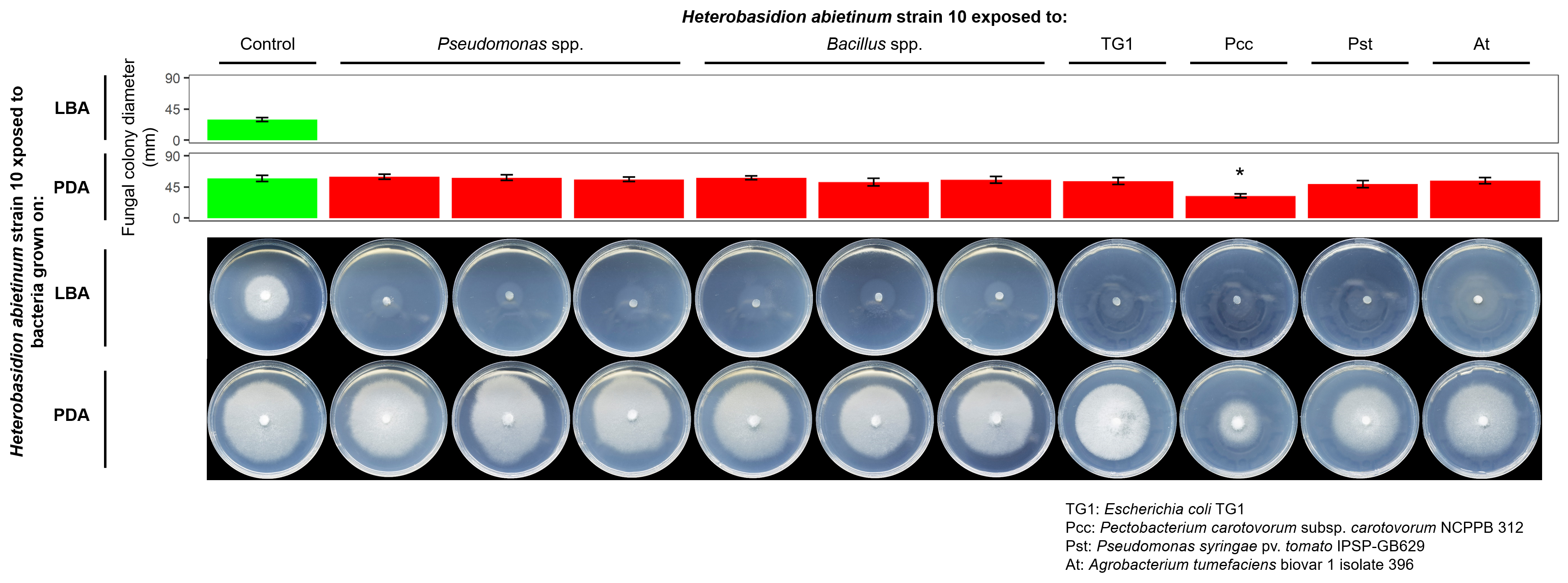

Supplement: Supplementary Figure 1 — Phylogenetic analysis of 63 isolates belonging to the Heterobasidion genus, including the strain 10 used in this study, computed by the maximum likelihood method and based on four concatenated genes. The genes used were: glutathione-S-transferase 1 (GST1), elongation factor 1-α (EFA), glyceraldehyde 3-phosphate dehydrogenase (G3P), and a transcription factor (TF). The percentage of trees (out of 1000 bootstraps) in which the associated taxa clustered together is shown next to the branches. There were a total of 1171 sites in the final dataset. The tree is drawn to scale, with branch lengths measured in the number of substitutions per site. The accession numbers of the isolates are given in Supplementary Table 1. [file Presentation_1.zip › Supplementary material/Supplementary Figure 5.TIF]

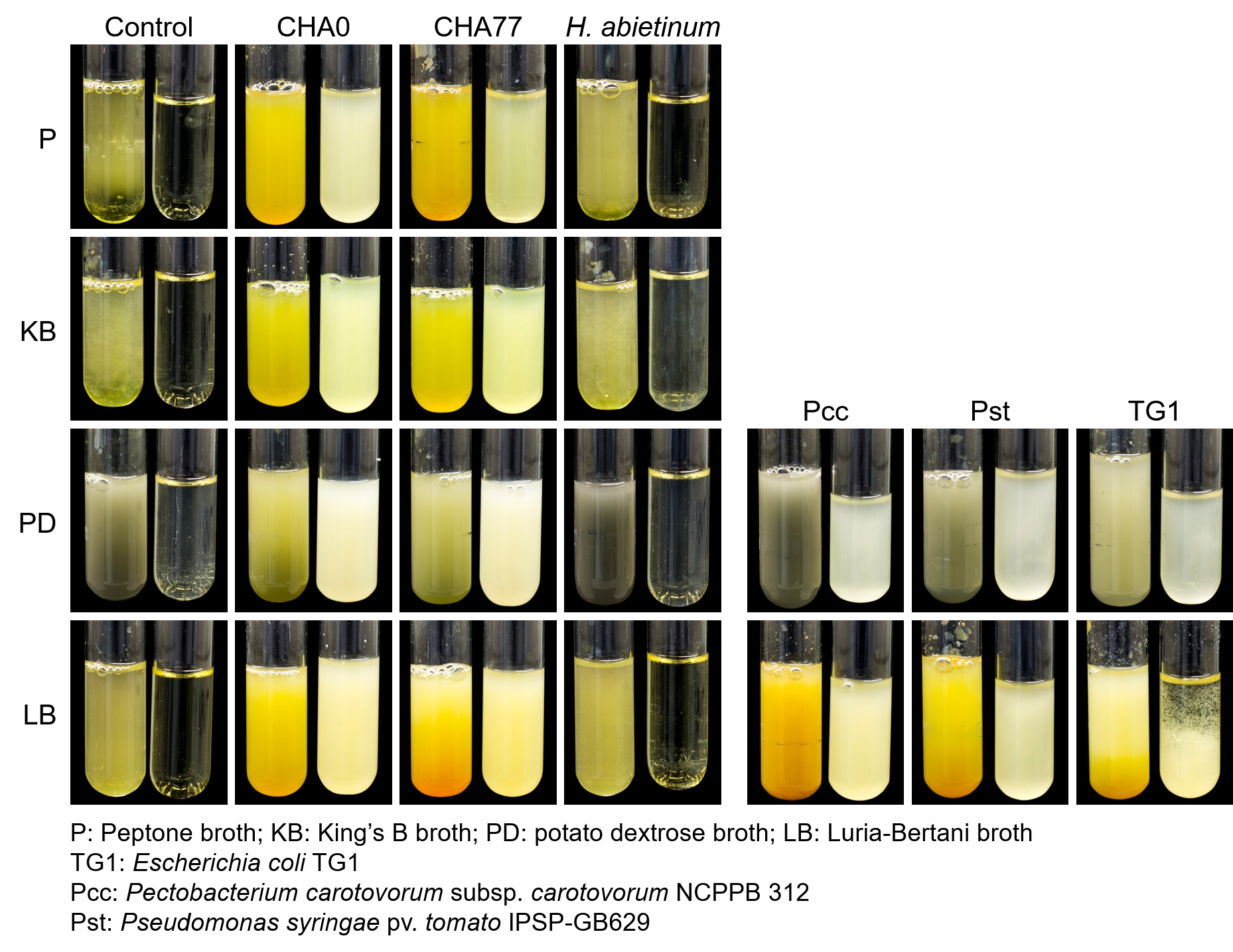

Supplement: Supplementary Figure 1 — Phylogenetic analysis of 63 isolates belonging to the Heterobasidion genus, including the strain 10 used in this study, computed by the maximum likelihood method and based on four concatenated genes. The genes used were: glutathione-S-transferase 1 (GST1), elongation factor 1-α (EFA), glyceraldehyde 3-phosphate dehydrogenase (G3P), and a transcription factor (TF). The percentage of trees (out of 1000 bootstraps) in which the associated taxa clustered together is shown next to the branches. There were a total of 1171 sites in the final dataset. The tree is drawn to scale, with branch lengths measured in the number of substitutions per site. The accession numbers of the isolates are given in Supplementary Table 1. [file Presentation_1.zip › Supplementary material/Supplementary Figure 6.TIF]

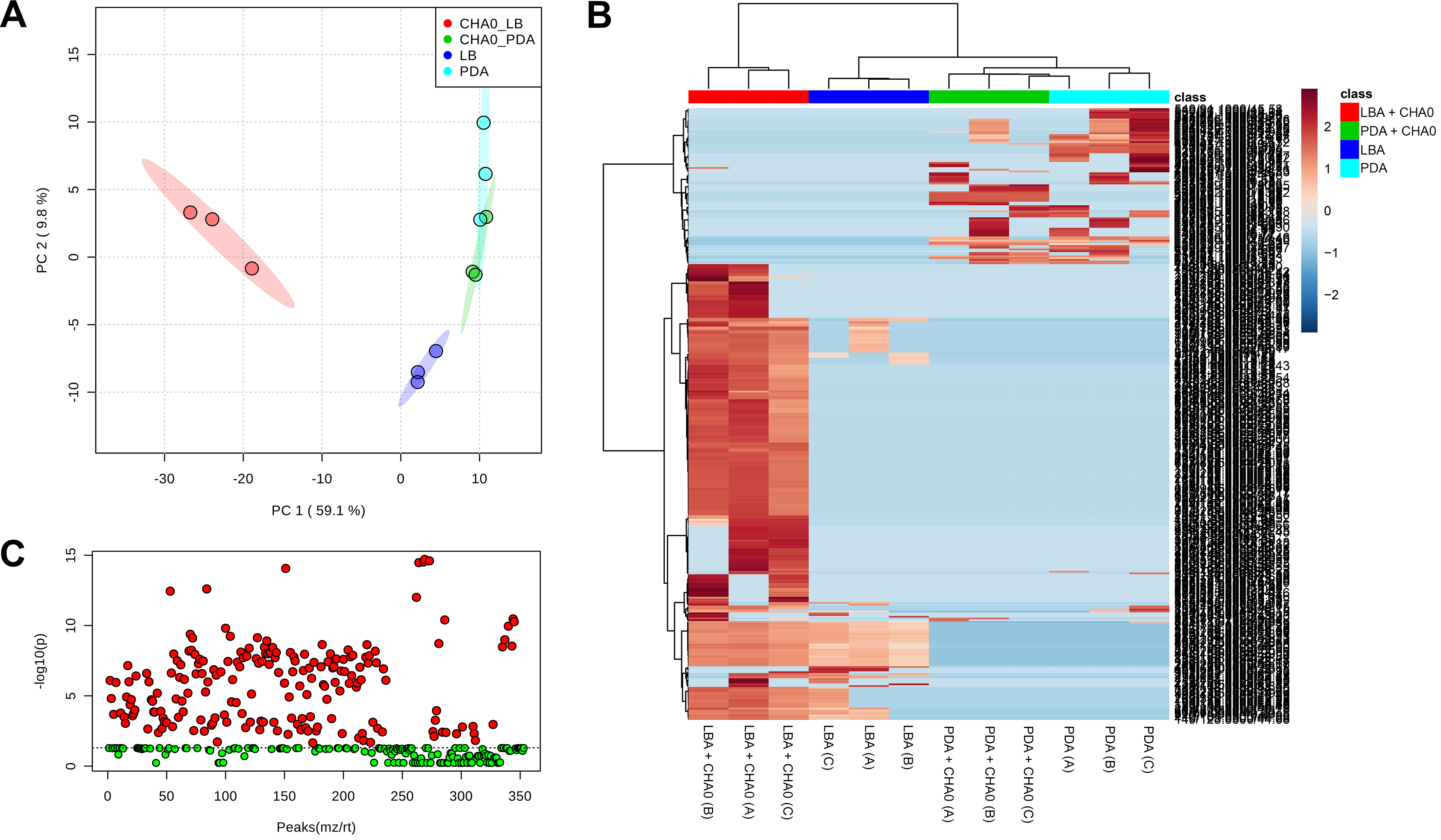

Supplement: Supplementary Figure 1 — Phylogenetic analysis of 63 isolates belonging to the Heterobasidion genus, including the strain 10 used in this study, computed by the maximum likelihood method and based on four concatenated genes. The genes used were: glutathione-S-transferase 1 (GST1), elongation factor 1-α (EFA), glyceraldehyde 3-phosphate dehydrogenase (G3P), and a transcription factor (TF). The percentage of trees (out of 1000 bootstraps) in which the associated taxa clustered together is shown next to the branches. There were a total of 1171 sites in the final dataset. The tree is drawn to scale, with branch lengths measured in the number of substitutions per site. The accession numbers of the isolates are given in Supplementary Table 1. [file Presentation_1.zip › Supplementary material/Supplementary Figure 7.TIF]

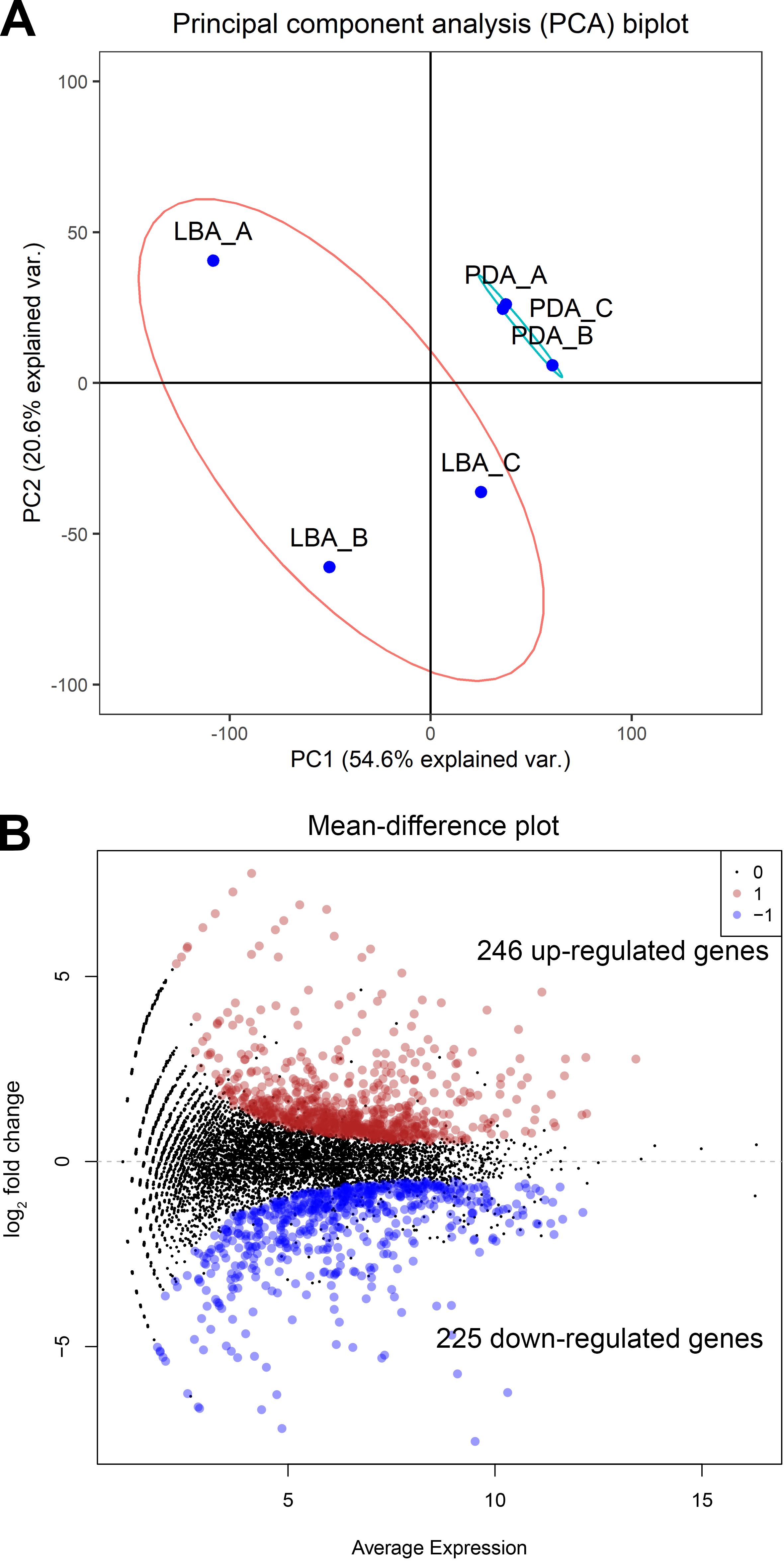

Supplement: Supplementary Figure 1 — Phylogenetic analysis of 63 isolates belonging to the Heterobasidion genus, including the strain 10 used in this study, computed by the maximum likelihood method and based on four concatenated genes. The genes used were: glutathione-S-transferase 1 (GST1), elongation factor 1-α (EFA), glyceraldehyde 3-phosphate dehydrogenase (G3P), and a transcription factor (TF). The percentage of trees (out of 1000 bootstraps) in which the associated taxa clustered together is shown next to the branches. There were a total of 1171 sites in the final dataset. The tree is drawn to scale, with branch lengths measured in the number of substitutions per site. The accession numbers of the isolates are given in Supplementary Table 1. [file Presentation_1.zip › Supplementary material/Supplementary Figure 8.TIF]

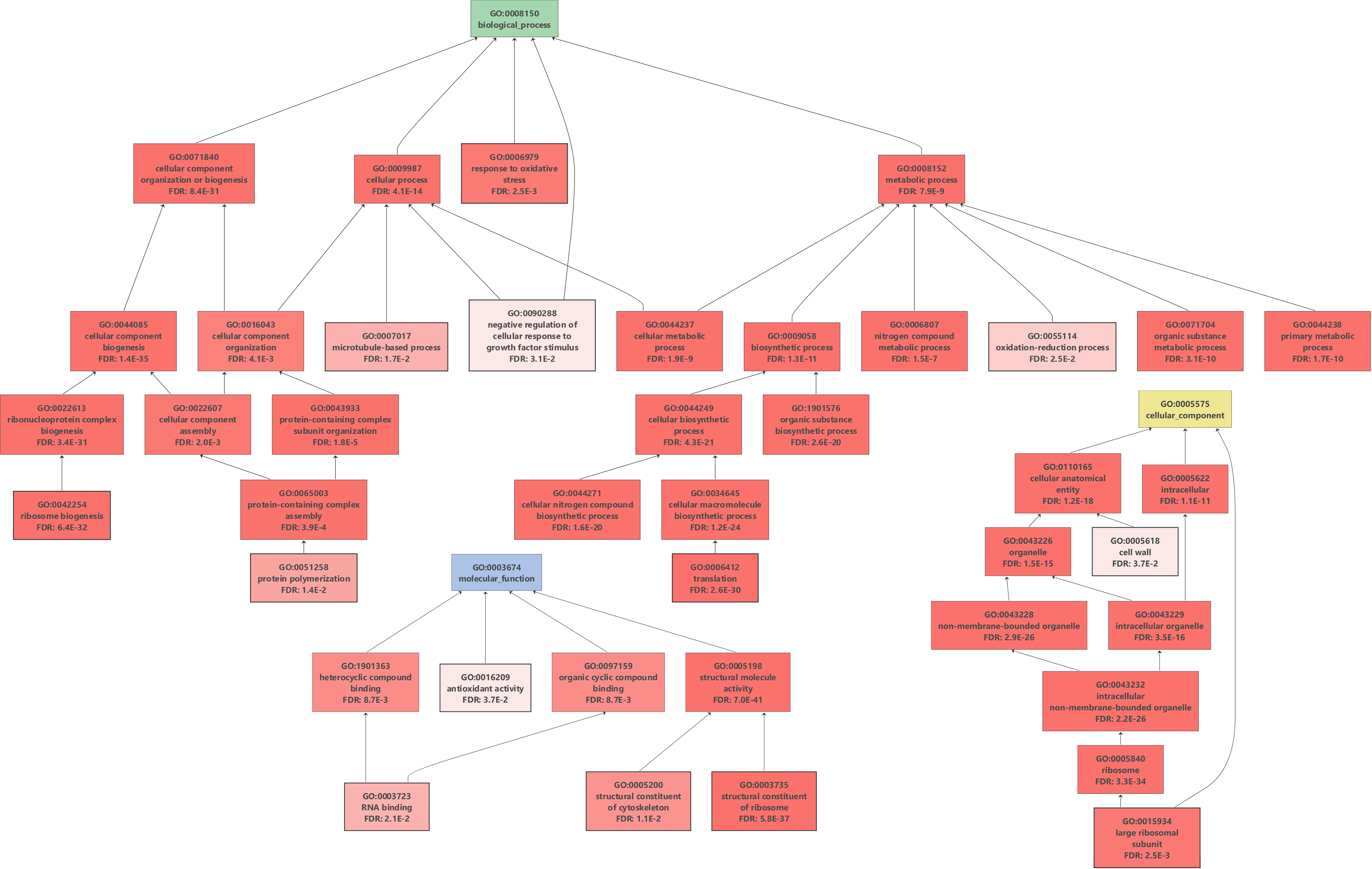

Supplement: Supplementary Figure 1 — Phylogenetic analysis of 63 isolates belonging to the Heterobasidion genus, including the strain 10 used in this study, computed by the maximum likelihood method and based on four concatenated genes. The genes used were: glutathione-S-transferase 1 (GST1), elongation factor 1-α (EFA), glyceraldehyde 3-phosphate dehydrogenase (G3P), and a transcription factor (TF). The percentage of trees (out of 1000 bootstraps) in which the associated taxa clustered together is shown next to the branches. There were a total of 1171 sites in the final dataset. The tree is drawn to scale, with branch lengths measured in the number of substitutions per site. The accession numbers of the isolates are given in Supplementary Table 1. [file Presentation_1.zip › Supplementary material/Supplementary Figure 9.TIF]
